# Ensemble‐based classification using microRNA expression identifies a breast cancer patient subgroup with an ultralow long‐term risk of metastases

**DOI:** 10.1002/cam4.7089

**Published:** 2024-04-27

**Authors:** Ines Block, Mark Burton, Kristina P. Sørensen, Martin J. Larsen, Thi T. N. Do, Martin Bak, Søren Cold, Mads Thomassen, Qihua Tan, Torben A. Kruse

**Affiliations:** ^1^ Department of Clinical Genetics Odense University Hospital Odense Denmark; ^2^ Human Genetics, Department of Clinical Research University of Southern Denmark Odense Denmark; ^3^ Clinical Genome Center University of Southern Denmark and Region of Southern Denmark Odense Denmark; ^4^ Department of Pathology Odense University Hospital Odense Denmark; ^5^ Department of Pathology Hospital of Southwest Jutland Esbjerg Denmark; ^6^ Department of Oncology Odense University Hospital Odense Denmark; ^7^ Epidemiology, Department of Public Health University of Southern Denmark Odense Denmark; ^8^ Present address: Department of Mathematics and Computer Science University of Marburg Marburg Germany

**Keywords:** low‐risk breast cancer, lymph node negative, microRNA‐based recurrence prediction, overtreatment reduction, systematically untreated

## Abstract

**Background:**

Current clinical markers overestimate the recurrence risk in many lymph node negative (LNN) breast cancer (BC) patients such that a majority of these low‐risk patients unnecessarily receive systemic treatments. We tested if differential microRNA expression in primary tumors allows reliable identification of indolent LNN BC patients to provide an improved classification tool for overtreatment reduction in this patient group.

**Methods:**

We collected freshly frozen primary tumors of 80 LNN BC patients with recurrence and 80 recurrence‐free patients (mean follow‐up: 20.9 years). The study comprises solely systemically untreated patients to exclude that administered treatments confound the metastasis status. Samples were pairwise matched for clinical‐pathological characteristics to minimize dependence of current markers. Patients were classified into risk‐subgroups according to the differential microRNA expression of their tumors via classification model building with cross‐validation using seven classification methods and a voting scheme. The methodology was validated using available data of two independent cohorts (*n* = 123, *n* = 339).

**Results:**

Of the 80 indolent patients (who would all likely receive systemic treatments today) our ultralow‐risk classifier correctly identified 37 while keeping a sensitivity of 100% in the recurrence group. Multivariable logistic regression analysis confirmed independence of voting results from current clinical markers. Application of the method in two validation cohorts confirmed successful classification of ultralow‐risk BC patients with significantly prolonged recurrence‐free survival.

**Conclusion:**

Profiles of differential microRNAs expression can identify LNN BC patients who could spare systemic treatments demanded by currently applied classifications. However, further validation studies are required for clinical implementation of the applied methodology.

## Abstract 2

Our study shows that differential microRNA expression analysis has the potential to identify a subgroup of Breast cancer (BC) patients with an extremely low long‐term risk of metastases. The ensemble‐based classification approach developed could form the starting point for a diagnostic tool to avoid unnecessary overtreatment of a significant proportion of BC patients.

## 1 INTRODUCTION

Breast cancer (BC) is the second most common cancer type in women and still associated with a mortality rate of approximately 13% in the global female population,^1^, ^2^ despite early detection through nationwide mammography programs and improved treatment options. Correct classification of patients and selection of optimal therapies remains a major challenge. Most patients do not die because of the primary tumor that is immediately removed after diagnosis, but because of metastasis to vital organs. However, the occurrence of metastases is difficult to predict at the time of diagnosis. To minimize the risk of BC recurrence, approximately 90% of all current BC patients are classified as high‐risk patients according to their clinical and histopathological characteristics. Consequently, these BC patients receive adjuvant systemic treatments, although up to 40% of these patients are unlikely to benefit from treatment and are likely to be cured by surgery and radiotherapy alone.^3^, ^4^ On the contrary, these patients may suffer from severe side‐effects and their overtreatment imposes significant costs on the healthcare system.

Thus, research currently focuses on optimizing therapies and reducing overtreatment. Unfortunately, there are currently no reliable markers that unambiguously can identify breast tumors that are not capable of forming life‐threatening metastases 5, 10, or 20 years after surgery.^4^, ^5^, ^6^, ^7^

Delahaye et al. and Esserman et al. applied an adjusted cutoff for the 70‐gene MammaPrint gene expression profile to identify a patient group with an “ultralow” risk of cancer recurrence.^5^, ^6^ Using formalin‐fixed paraffin‐embedded (FFPE) tissue, they classified 4.6% to 15% of all analyzed patients as ultralow‐risk patients, not benefitting from adjuvant endocrine treatments or chemotherapies.^5^, ^6^

Although encouraging, more efficient prognostic markers are required to further reduce overtreatment while ensuring that the rate of recurrence will not increase as a consequence of treatment reduction.

In comparison to numerous gene expression signatures,^5^, ^6^, ^7^, ^8^ so far no signature based on microRNAs has passed clinical trials although microRNAs are key regulators of gene expression, appear to be more stable than coding RNAs in preserved tissues, and, represent promising prognostic markers in BC.^9^, ^10^

Hence, we tested whether differential miRNA expression in primary tumors may predict recurrence (regional or distant metastasis) in systemically untreated BC patients who have no evidence of metastases at diagnosis. We developed an ensemble‐based classification strategy using our own data set and independently validated our method using published miRNA expression data.^11^, ^12^, ^13^ We also illustrate the advantage of the applied pair‐matched design by testing the independent performance of our method.

## 2 METHODS

### 2.1 Collection of tumor biopsies and patient characteristics

**FIGURE 1:**
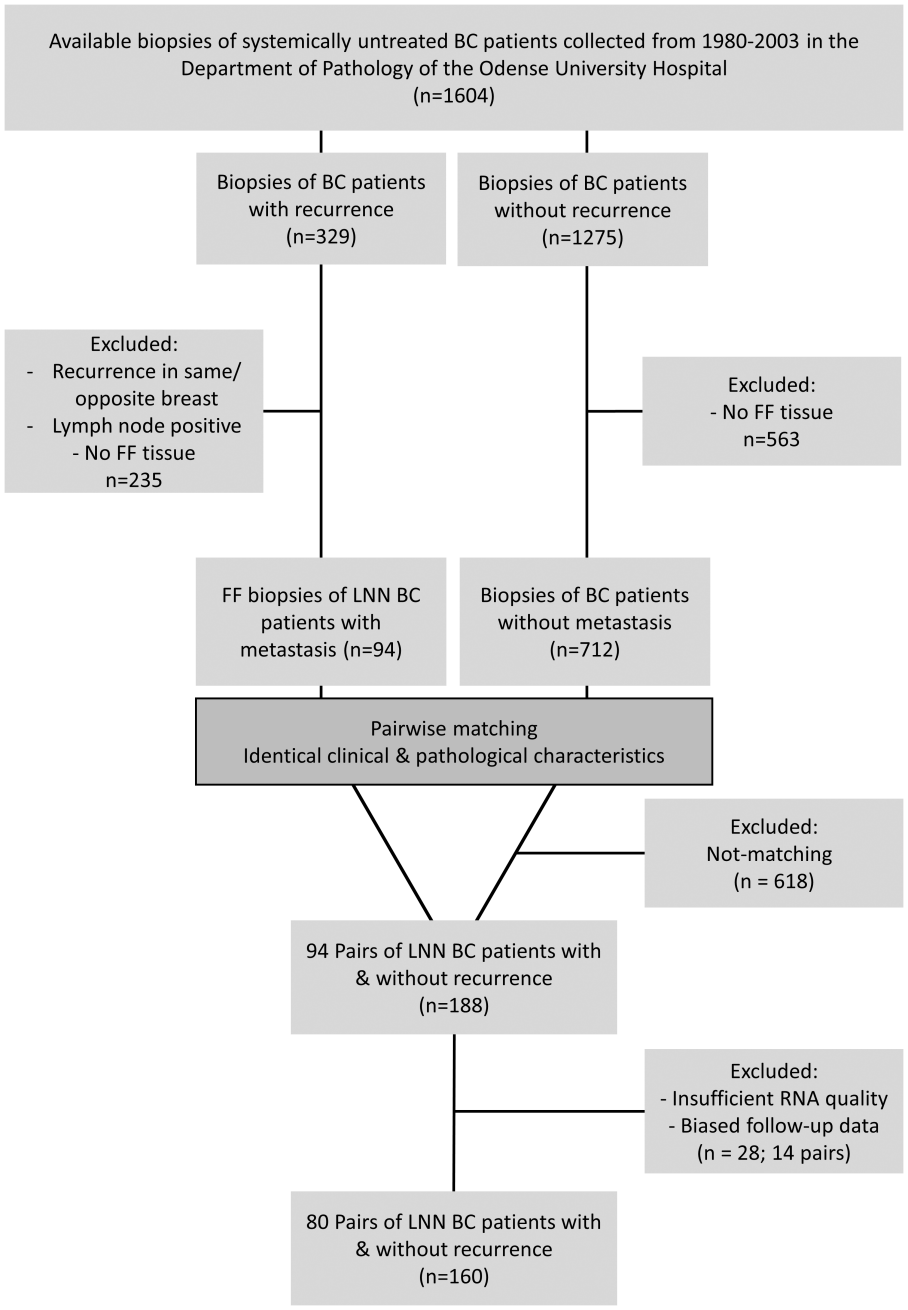
CONSORT diagram summarizing selection of 160 lymph node negative (LNN) patients diagnosed with invasive breast cancer (BC) for the paired study. In total 1604 biopsies of low‐risk breast cancer patients were available. All patients did not receive any systemic adjuvant treatment from 1980 to 2003 according to the criteria (see Table S1) of the Danish Breast Cancer Cooperative Group (DBCG). Fresh frozen (FF) biopsies of low‐risk patients with recurrence were matched with FF biopsies of recurrence‐free patients according to tumor type, year of surgery, tumor diameter, patient age, receptor status and histological grade.

We selected 160 fresh frozen tumor biopsies out of 1604 biopsies of lymph node negative (LNN) patients with diagnosed invasive BC who did not receive any systemic adjuvant treatment according to the guidelines (see Table S1) of the Danish Breast Cancer Cooperative Group (DBCG) from 1980 to 2003.^14^ Inclusion and exclusion criteria are outlined in Figure 1. Eighty patients developed regional or distant metastases within 10 years after diagnosis while 80 patients did not experience metastases (mean follow‐up of 20.9 years). Patient biopsies were matched according to tumor type, year of surgery, tumor diameter (range: 4–50 mm), patient age (range: 33–88 years), receptor status (ER, PR, and n/a) and histological grade (grade 1–3 or n/a) (Table 1).^15^

**TABLE 1:** Patient and tumor characteristics of the three cohorts of systemically untreated, lymph node negative breast cancer patients included in the study.

	OUH		D'Aiuto et al.^12^		METABRIC^11^, ^13^	
Number of patients (%)	160 (100)		123 (100)		339 (100)	
Metastasis	Yes	No	Yes	No	Yes	No
	80 (50)	80 (50)	59 (48)	64 (52)	78 (23)	261 (77)
Age at diagnosis						
≤50 years	14 (8.8)	10 (6.3)	18 (14.6)	27 (22)	27 (8)	68 (20.1)
>50 years	66 (41.3)	70 (43.8)	41 (33.3)	37 (30.1)	51 (15)	193 (56.9)
Tumor size						
<2 cm	27 (16.9)	30 (18.8)	18 (14.6)	23 (18.7)	34 (10.0)	119 (35.1)
2–5 cm	53 (33.1)	49 (30.6)	39 (31.7)	41 (33.3)	41 (12.1)	125 (36.9)
>5 cm	–	–	1 (0.8)	–	2 (0.6)	9 (2.7)
n/a	–	1 (0.6)	1 (0.8)	–	1 (0.3)	8 (2.4)
Estrogen receptor status^a^						
Positive	52 (32.5)	50 (31.3)	47 (38.2)	53 (43.1)	63 (18.6)	179 (52.8)
Negative	22 (13.8)	24 (15)	12 (9.8)	11 (8.9)	11 (3.2)	61 (18)
n/a	6 (3.8)	6 (3.75)	–	–	4 (1.2)	21 (6.2)
Tumor type						
Invasive ductual carcinoma (IDC)	62 (38.8)	65 (40.6)	48 (39)	49 (39.8)	60 (17.7)	169 (49.7)
Invasive lobular carcinoma (ILC)	9 (5.6)	9 (5.6)	9 (7.3)	3 (2.4)	7 (2.1)	18 (5.3)
Mucinous carcinoma	2 (1.3)	2 (1.3)	–	–	1 (0.3)	8 (2.4)
Papillary carcinoma	3 (1.9)	2 (1.3)	–	–		
Carcinoma with metaplasia	2 (1.3)	2 (1.3)	–	‐		
Mixed IDC/ILC	–	–	2 (1.6)	5 (4.1)	4 (1.2)	7 (2.1)
Other	–	–	–	7 (5.7)	5 (1.5)	52 (15.3)
n/a	2 (1.3)	–	–	–	1 (0.3)	7 (2.1)
Histologic grade						
1 (good)	12 (7.5)	15 (9.4)	n/a	n/a	7 (2.1)	33 (9.7)
2 (intermediate)	28 (17.5)	25 (15.6)	n/a	n/a	44 (13)	114 (33.6)
3 (poor)	22 (13.8)	24 (15)	n/a	n/a	22 (6.5)	87 (25.7)
n/a	18 (11.3)	16 (10)	n/a	n/a	5 (1.5)	27 (8)
Mean time to metastasis (months)	58.5	n/a	30.5	n/a	n/a	n/a
Mean follow‐up (months)	88.3	250.35	n/a	118	103.03	128.34
Alive at end of follow‐up	1	48	n/a	n/a	16	181

Abbreviation: n/a, not available/applicable.

^a^ As defined by immunohistochemistry.

We introduced this paired study design because matching the primary tumors ensured the independence of classical clinical or pathological characteristics and minimized bias related to sample retrieval, storage, and diagnostic procedures. All clinical‐pathological information used for sample matching was extracted from the DBCG database, the Funen pathology database, or the nationwide pathology database as approved by the Danish National Committee for Health Research (S‐VF‐20020142). No informed consent was obtained from the patients involved in this retrospective study as approved by the Ethical Committee. Patients who objected to the use of their tumor material for research purposes through the Danish Tissue Utilization Register were excluded from the study.

### 2.2 RNA extraction and microRNA profiling

Total RNA was isolated and miRNA expression was analyzed as previously described.^16^ Expression data have been deposited in NCBI's Gene Expression Omnibus^17^ and are accessible through GEO Series accession numbers GSE126125 and GSE103161 (https://www.ncbi.nlm.nih.gov/geo/query/acc.cgi).

### 2.3 Validation data acquisition

Publicly available miRNA expression data published by D'Aiuto et al.^12^ were collected from NCBI's Gene Expression Omnibus database (accession number: GSE59829).^17^ Expression data published by Dvinge et al.^11^ and Curtis et al. (METABRIC)^13^ were retrieved from the European Phenome‐Genome Archive (https://www.ebi.ac.uk/ega/home)^18^ after access was granted by METABRIC. Clinical‐pathological characteristics of patients and tumor samples are summarized in Table 1.

### 2.4 Data processing, classification, and voting

Scanning of the arrays and data preprocessing were performed as previously described.^16^, ^19^

**FIGURE 2:**
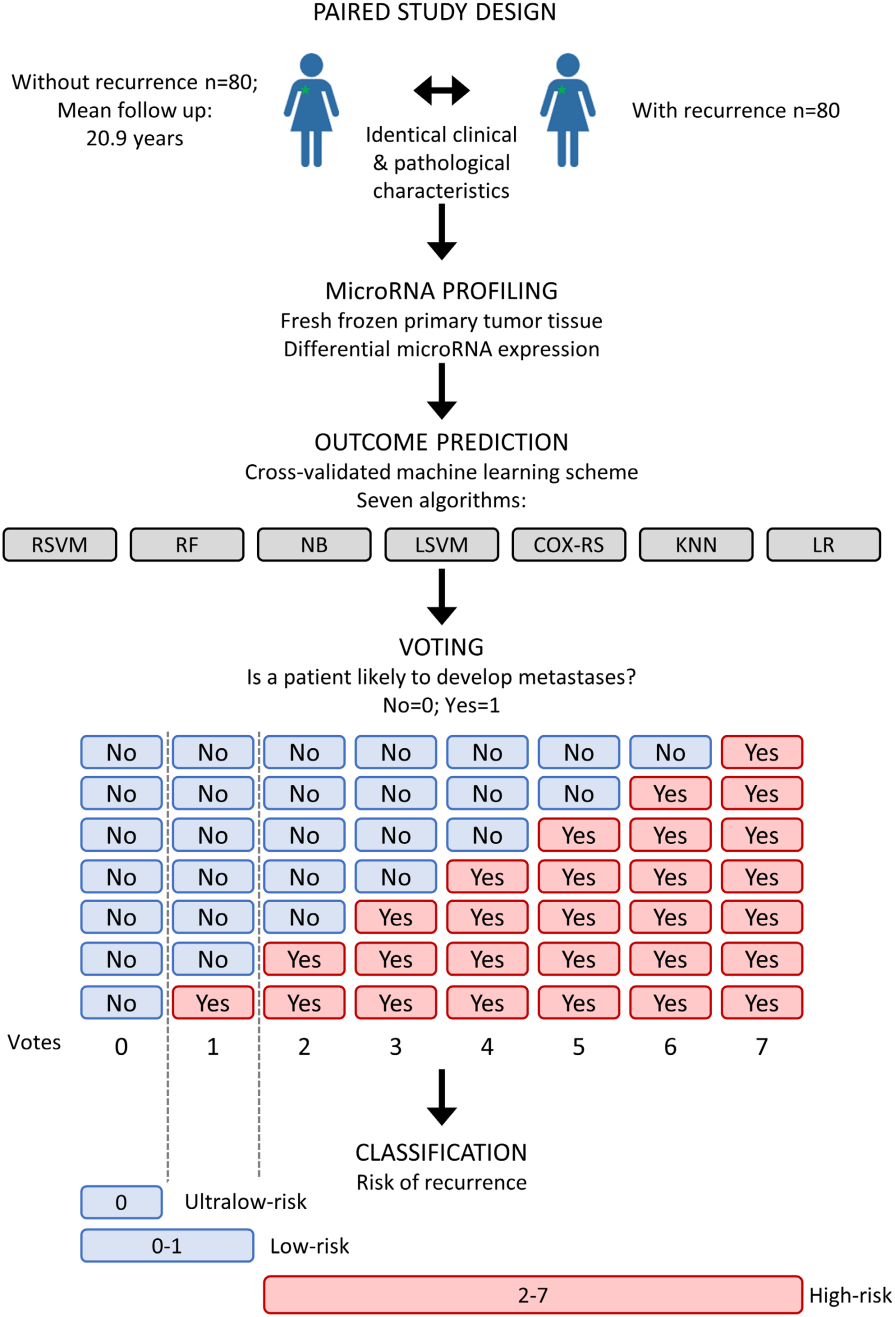
Study design and methodological work‐flow. We analyzed 160 freshly frozen primary tumors from systemically untreated, lymph node negative patients diagnosed with invasive BC. Eighty patients developed metastases within 10 years and were matched according to classical pathological and clinical characteristics with 80 tumors of patients who did not experience recurrence (mean follow‐up of 20.9 years). Differential miRNA expression was analyzed to predict outcome using a cross‐validated machine learning routine (Figure S1) and seven classification methods (RSVM, RF, NB, LSVM, COX‐RS, KNN, and LR). Each classification method voted if a patient is likely (vote = 1) or not likely (vote = 0) to develop a metastasis based on the calculated probability of recurrence (RSVM, RF, NB. LSVM and LR) or Cox‐risk regression sum (COX‐RS) or class assignment (KNN). Patients receiving 0 votes were defined as having an ultralow‐risk of recurrence, while the patient group receiving 0–1 votes were defined as low‐risk patients.

In this group of low‐risk patients poor clinical outcome is fairly rare. Therefore, our paired study design increases power of the analysis by enriching for clinical endpoints in comparison to cohort studies. Furthermore, our balanced design allows for identifying expression profiles independent of classical markers.^15^ Classification of samples belonging to either the recurrence or recurrence‐free group was conducted using seven machine learning (ML) methods: Radial‐based kernel Support Vector Machine (RSVM), random forest (RF), Naïve Bayes (NB), Linear kernel Support Vector Machine (LSVM), COX risk score (COX‐RS), K‐Nearest Neighbor (KNN), and Logistic Regression (LR). The methods were combined with a leave‐one‐pair out cross‐validation (Data S1–Supplementary Methods, Figure S1).^20^, ^21^ We applied a voting scheme (Figure 2) that has been shown to be advantageous for classifying BC patients based on expression data to further strengthen our classification.^22^

We further developed miRNA profiles derived by the seven ML methods from our dataset, and tested their individual performance in two external validation datasets (Figure S1) by applying the procedures described above. An extended description of all data processing steps, leave‐one‐pair‐out cross‐validation, feature selection, statistical analysis, and code availability is provided in the Supplement (Data S1—Supplementary Methods).

## 3 RESULTS

### 3.1 Biopsy selection and study design

We identify conserved tumor material of a prospectively collected cohort of LNN patients with invasive BC.^14^ Out of 1604 BC patients 329 patients (20.5%) experienced recurrence while 1275 patients (79.5%) remained metastasis‐free (Figure 1) although none received systemic adjuvant treatments according to the DBCG guidelines^14^ (see Table) from 1980 to 2003. We introduced a paired study design^15^ (Figure 2) and selected freshly frozen biopsies of 80 patients who developed regional or distant metastases and biopsies of 80 patients with matching clinical and pathological characteristics (Table 1) who did not experience metastases (mean follow‐up of 20.9 years).

### 3.2 Classification of patients

We analyzed the microRNA expression in the 160 primary tumor samples and identified differential expressed microRNAs with a false discovery rate (FDR) below 5%. The subsequent classification by seven individual ML methods (Figure 2) divided our patients into subgroups with a high‐risk and a low‐risk of recurrence with an overall classification accuracy ranging from 77% (76% sensitivity, 78% specificity; *p*= 4.96 × 10^−12^) for the COX‐RS to an accuracy of 86% (84% sensitivity, 88% specificity; *p* = 9.84 × 10^−22^) for the RF‐based classification (Table S2; Figure S2). The ML methods were combined with a leave‐one‐pair‐out cross‐validation (Figure S1A, Data S1—Supplementary Methods) which provides an unbiased performance estimate and has proven to be optimal for the analysis of smaller datasets.^20^, ^21^ In brief, a single pair of matched samples served as test samples and the remaining samples as a training set. This was repeated until all pairs had been left out once whereby in each round a different set of the 217 differentially expressed microRNAs served as features for the classification of the test samples. The accuracy of the classifier was determined by the correctly classified samples.

**FIGURE 3:**
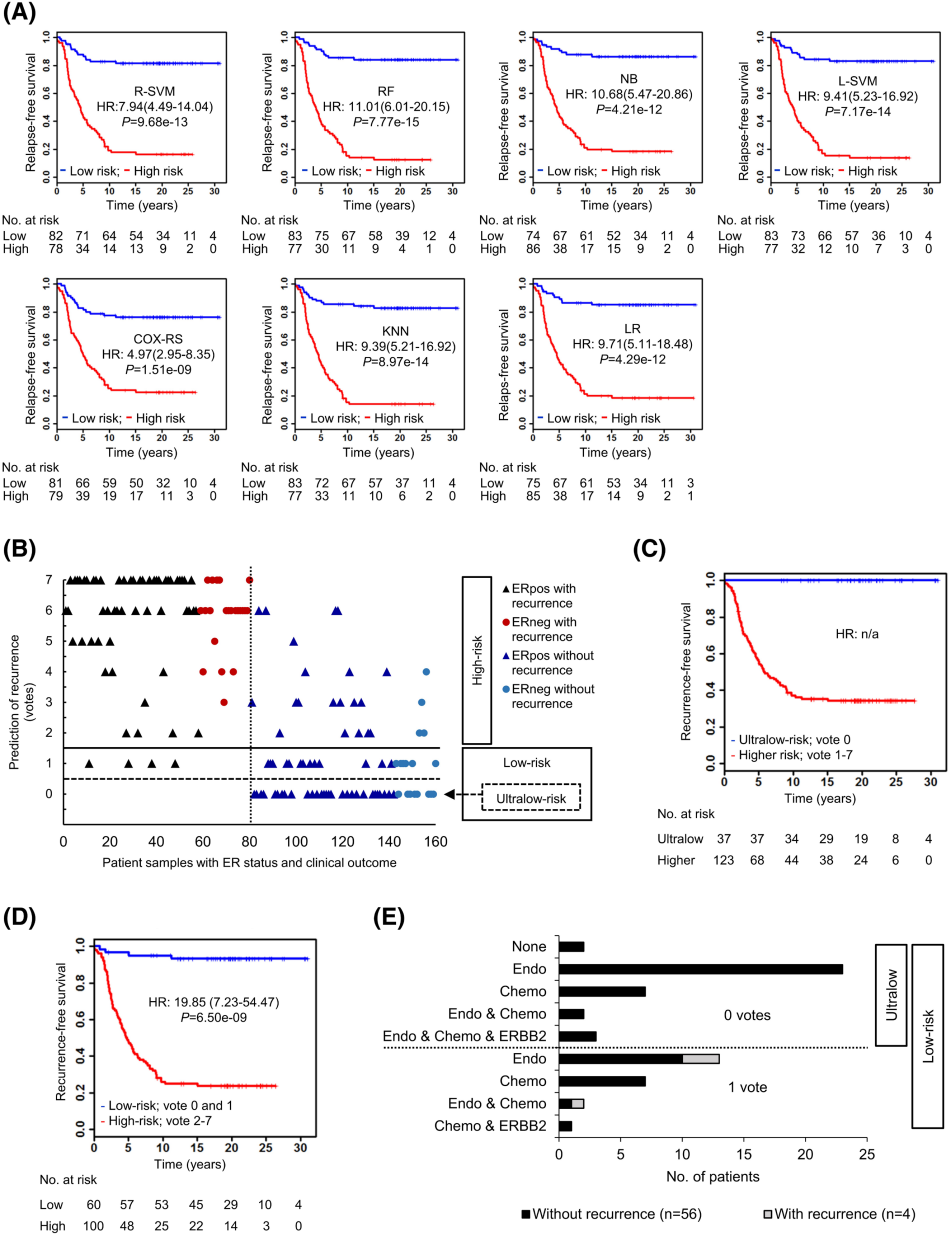
Classification and survival analysis. (A) Survival analysis using seven classification methods (RSVM, RF, NB, LSVM, COX‐RS, KNN, and LR). Kaplan–Meier survival curves demonstrate the model based predicted recurrence‐free survival. HR: hazard ratios with 95% confidence interval (in parentheses) are displayed. Significance of the HR was tested by Cox proportional hazards regression model (*p*‐value). (B) Dot plot summarizing voting results illustrating the predicted votes versus the diagnosed occurrence of metastases in estrogen receptor positive (ERpos) and negative (ERneg) patients. The dashed vertical line separates the patients with recurrence (left handed) from the recurrence‐free patients (right handed). The horizontal line discriminates patients with a predicted low‐risk (0–1 votes) of recurrence from those with a high risk of recurrence (2–7 votes). The dashed horizontal line further separates patients with an ultralow‐risk (0 votes) of recurrence from the remaining patients. Kaplan–Meier curves of recurrence‐free survival according to model based prediction for ultralow‐risk patients (C) and low‐risk patients (D). HR: hazard ratios with 95% confidence interval (in parentheses) are displayed. Significance of the HR was tested by Cox proportional hazards regression model (*p*‐value). (E) Recommended treatment regimens for predicted ultralow and low‐risk patients if they would have been diagnosed in Denmark today whereby treatment selection is based on available clinical and pathological information. Chemo, chemotherapy; Endo, ER targeted treatment; ERBB2, ERBB2/HER2 targeted treatment; None, no treatment.

Kaplan–Meier survival curves demonstrate significant differences in recurrence‐free survival for each method between patients with low and high‐risk of recurrence (Figure 3A). The results emphasize the general potential of the differentially expressed microRNAs to identify low‐risk patients. However, we tried to improve our classification by combining individual results of the seven ML methods.

Thus, we applied a voting scheme (Figure 2) to define the cumulative risk of recurrence for individual patients calculated by all seven methods to improve our classification.^22^ The corresponding voting results range from 0 to 7 whereby 2–7 votes indicate a high risk, 0–1 votes a low‐risk, and 0 votes an ultralow‐risk for metastases. Applying these criteria, we predicted for 37 out of 160 patients an ultralow‐risk (0 votes, Figure 3B) and for 60 out of 160 patients a low‐risk of recurrence (0 and 1 vote, Figure 3B). Among the 37 ultralow‐risk patients, none experienced recurrence according to our follow‐up data while among the 60 low‐risk patients, 56 patients remained cured after surgery. Correspondingly, these patients had significantly prolonged recurrence‐free survival expectations in comparison to the remaining patients as determined by Kaplan–Meier survival analysis (Figure 3C,D). For the ultralow‐risk group hazard ratios (HR) could not be calculated due to the lack of incidences in this group. For the low‐risk group, a HR of 19.85 with a 95% confidence interval (CI) ranging from 7.23 to 54.47 was determined (*p* = 6.5e^−09^). In comparison, in the Kaplan–Meier survival analysis, HR of the individual ML methods were lower with values between 4.97 and 11.01 (Figure 3A). Notably, according to the current classification guidelines (Tables S3 and S4) only two of the ultra‐low classified patients would remain systemically untreated if they would be diagnosed in Denmark today (Figure 3E).

The voting results were independent of any classical markers currently applied in the clinics as verified by multivariable logistic regression analysis (Table 2). Although estrogen receptor (ER) expression is considered a critical factor for outcome prediction and patients with low ER expression are commonly assigned to chemotherapy, our microRNA‐based classification strategy was independent of the ER status (Table 2). Moreover, of 40 ER negative patients, 14 (35%) were classified as low‐risk (Figure S3; Table S5) all belonging to the 22 ER negative and recurrence‐free patients, that is none of the ER negative patients with recurrence were misclassified as low‐risk.

**TABLE 2:** Multivariable logistic regression analysis testing independence of voting results from classical clinical variables.

	Ultralow‐risk			Low‐risk		
	OR	CI	*p*	OR^a^	CI	*p*
ER status	1.20	0.32–4.58	0.78	1.70	0.47–6.16	0.42
Age	1.67	0.45–6.19	0.44	1.12	0.31–4.08	0.86
Tumor size	2.53	0.90–7.12	0.078	1.61	0.60–4.36	0.35
Tumor grade	1.38	0.40–4.79	0.61	2.50	0.75–8.39	0.14
Outcome	n/a	n/a	n/a	41.81	12.69–137.76	8.44 × 10^−10^

Abbreviations: CI, 95% confidence interval; ER, estrogen receptor; n/a, not applicable; OD, odds ratio; *p*, *p*‐value of logistic regression analysis.

### 3.3 Validation using independent data

While ample gene expression data are accessible, only limited data are available on primary tumors of systemically untreated patients with long follow‐ups. Even rarer are microRNA expression data which often vary substantially due to technical differences.^11^, ^12^, ^13^, ^23^, ^24^ We identified two suitable datasets comprising 123 (D'Aiuto et al.) and 339 (METABRIC) systemically untreated BC patients.^11^, ^12^, ^13^ Unfortunately, only 859 and 815 mature microRNAs were analyzed in the respective studies, in comparison to 1212 mature microRNAs analyzed in our cohort (OUH). Moreover, after proper correction for multiple testing, 217 microRNAs were significantly deregulated in our dataset while no microRNA in the validation sets reached a false discovery rate (FDR) below 5%. Despite these technical differences, we selected the most informative set of microRNAs for each ML method using our OUH cohort for preselection and performed patient classification via cross‐validation in the D'Aiuto et al. and METABRIC cohort (Figure S1B, Data S1—Supplementary Methods). Since not all microRNAs were analyzed in these independent datasets, reduced microRNA sets were subsequently used for outcome prediction by the seven individual ML methods. The individual sets per method selected based on all microRNAs analyzed are listed in Table S7. To improve the classification, we again applied our voting strategy. Applying a dataset‐specific cut‐off for voting, subgroups of patients with ultralow (0 votes for METABRIC; 2 votes for D'Aiuto et al.) and low (0 + 1 vote for METABRIC; 2 + 3 votes for D'Aiuto et al.) risk of recurrence could be identified (Table 3; Tables S5 and S6) which were significantly associated with prolonged recurrence‐free or overall survival (Figure S4). For example, 16 (13%) out of 123 patients in the D'Aiuto et al. dataset were predicted to have an ultralow‐risk for metastases. None of these patients experienced recurrence according to the available follow‐up data. Among the predicted 64 low‐risk patients 14 (22%) recurrences occurred. However, significant differences concerning the number of patients with and without recurrence who were allocated to the ultralow‐risk (*p* = 1.03 × 10^−5^, Table 3) or low‐risk group (*p* = 1.01 × 10^−9^, Table 3) confirm a non‐random classification.

**TABLE 3:** Overview of prediction performance using voting for patients without (wo rec) and with recurrence (w rec) classified as ultralow or low‐risk patients.

Dataset	Total number of patients	Number of patients wo rec correctly classified as ultralow‐risk (%)	Number of patients w rec incorrectly classified as ultralow‐risk (%)	*p*‐value*	Number of patients wo rec correctly classified as low‐risk (%)	Number of patients w rec incorrectly classified as low‐risk (%)	*p*‐value*
		Ultralow‐risk			Low‐risk		
OUH	160	37/80 (46)	0/80 (0)	3.05 × 10^−14^	56/80 (70)	4/80 (5)	4.32 × 10^−19^
D'Aiuto et al.^a^	123	16/64 (25)	0/59 (0)	1.03 × 10^−5^	50/64 (78)	14/59 (23)	1.01 × 10^−9^
METABRIC	339	3/261 (1.2)	1/78 (1)	0.77	35/261 (13)	3/78 (4)	0.011

Abbreviations: w rec, with recurrence; wo rec, without recurrence.

^a^ The ultralow‐risk group was defined as patients receiving 2 votes and the low‐risk group as patients receiving 2 or 3 votes as no patient received 0 or 1 votes (see Figure S4).

* *p*‐value determined by Fisher's exact test, one‐tailed.

## 4 DISCUSSION

Voting results of seven classification methods applied to differential microRNA expression in primary tumors of LNN women identified patients with a low and even ultralow‐risk of recurrence without any systemic treatment up to 20 years after diagnosis. The use of a paired study design allowed us to develop a prediction strategy that was independent of all markers currently used in the clinic, identifying ultralow and low‐risk patients with ER negative, ERBB2 positive tumors and tumor diameters larger than 2 cm. Comprehensive survival data collected 40 years ago, when patients usually received only surgery and radiotherapy, suggest that 25%–40% of all BC patients may survive without systemic adjuvant treatment.^3^, ^25^ However, the percentage of diagnosed patients not receiving systemic treatments in Western industrialized countries is remarkably lower although it must be assumed that the number of ultralow‐risk patients is even higher today due to national mammographic screening programs.^26^ For example, it is predicted that 60%–70% of 55‐year old BC patients with grade 2 tumors of 30 mm in diameter would survive the first 15 years post diagnosis with surgery alone (https://www.predict.nhs.uk/tool, version 2.1).^27^ Today, all of these patients are likely receiving systemic adjuvant treatments to reduce mortality in this subgroup^28^, ^29^ but only one in eight treated patients may benefit.^27^ On the contrary, the majority of patients may suffer from unnecessary side‐effects. In a recent study, Cardozo et al. analyzed the outcome of patients enrolled in the MINDACT trial and identified 15% of all patients as ultralow‐risk patients with an 8‐year distant metastasis free interval of 97%.^30^ However, only 16% of the identified ultralow‐risk patients did not receive any systemic treatment emphasizing the need for development of classification tools in systemically untreated patients for overtreatment reduction.^6^, ^7^, ^26^, ^30^, ^31^ However, the development of suitable markers requires analysis of tumor samples from systemically untreated patients with long follow‐up and detailed clinical and pathological data including a solid number of tumor samples from patients who develop metastases, which are underrepresented among systemically untreated LNN patients. The availability of long follow‐up data is particularly important to assess the need for endocrine treatments for ER‐positive patients, as late recurrence is very likely in this subgroup.^7^, ^32^ Moreover, analysis of freshly frozen tissues is often advantageous in initial exploratory phases because of better marker preservation. Consequently, collecting suitable samples is a challenge and a limiting factor for the development of novel markers aiming at reducing overtreatment.

In 2002 van't Veer and colleagues firstly applied expression profiling on 98 freshly frozen tumors from BC patients, most of whom had not received adjuvant systemic treatment and were followed up for a reasonable time span.^33^ They developed a 70 gene expression score separating high‐risk patients from low‐risk BC patients not benefitting from adjuvant systemic treatments.^33^, ^34^ The resulting MammaPrint expression score has also proven to identify minor patient subgroups with ER positive tumors that may have excellent long‐term survival prognosis without any systemic treatment.^5^, ^6^, ^30^ Ohnstad et al. identified 124 low‐risk patients among 231 hormone receptor positive, ERBB2 negative, LNN, and systemically untreated BC patients using the PAM50 risk of recurrence score.^8^ Five of these low‐risk patients died due to BC within 15 years after the initial diagnosis.^8^ Sjöström et al. developed a signature comprising 141 mRNAs and identified a low‐risk patient subgroup (*n* = 114) among 454 systemically untreated, postmenopausal, LNN patients with ER positive, and ERBB2 negative tumors.^7^ Out of 402 patients who remained recurrence‐free for 15 years, 108 (27%) were correctly classified as low risk, whereas 6 (12%) out of 52 patients with recurrence were misclassified as low risk.^7^

We developed an ensemble‐based classification strategy using matched patient cohorts with long follow‐up data. In our study out of 80 patients without recurrence after an average of over 20 years 37 (46%), were classified as ultralow‐risk and none out 80 patients who developed metastasis were misclassified. Using a slightly less stringent low‐risk classifier, 70% of patients without recurrence were correctly classified, but at the cost of 5% misclassifications in the metastasis‐group. The current study thus provides proof‐of‐concept results indicating a predictive role of microRNAs and even suggests that our method identifies a higher percentage of overtreated patients than mRNA‐based approaches.

A total of 217 significantly deregulated and clinically informative microRNAs formed the basis for our combined classification method, with a selected number of these microRNAs each entering the analysis by the seven individual ML approaches. To account for the smaller number of microRNAs analyzed in the independent validation datasets, the feature selection approach used determined optimal microRNA sets based on all microRNAs analyzed, comprising 147 individual microRNAs. Six differentially expressed microRNAs (hsa‐miR‐212‐3p, MIMAT0000269; hsa‐miR‐499‐5p, MIMAT0002870; hsa‐miR‐525‐5p, MIMAT0002838; hsa‐miR‐519e‐5p, MIMAT0002828; hsa‐miR‐640, MIMAT0003310; and hsa‐miR‐942‐5p MIMAT0004985) were repetitively used by all methods during the classification of the OUH patients. For five of these six microRNAs a predictive or functional role in BC tumorigenesis had already been described,^35^, ^36^, ^37^, ^38^, ^39^, ^40^, ^41^ but they had previously not been directly linked to risk of recurrence. However, only hsa‐miR‐212‐3p, hsa‐miR‐499‐5p, and hsa‐miR‐525‐5p were analyzed in the validation studies.

Although validation of our results was hindered due to the limited availability of suitable validation data, significant findings in two independent cohorts may underscore the potential of the miRnome for outcome prediction. Fewer low‐ and ultra‐risk patients were identified and more false positives were assigned in the validation cohorts, but neither the same method was used to quantify miRNA expression, nor were all miRNAs analyzed which played a strong predictive role in our dataset. Given these differences, it may not be surprising that no optimal validation could be performed.^24^ It may be a prerequisite to analyze FFPE tissues for further validation. Since microRNAs are considered to be very stable in FFPE tissue^9^ and such an analysis would allow the application of identical technical settings, we are convinced that transferring the applied strategy would produce predictive microRNA signatures.

In summary, we studied the microRNA expression in freshly frozen tumor samples of systemically untreated LNN patients with diagnosed invasive BC and extensive follow‐up records. By using a paired study design, a cross‐validated ML methodology, and voting, we were able to identify a subgroup of BC patients with a minimal long‐term risk for developing metastases. The majority of these patients would unnecessarily receive systemic treatments based on the currently applied classification. However, further studies are required for clinical implementation of the applied methodology.

## AUTHOR CONTRIBUTIONS

**Ines Block:** Conceptualization (equal); data curation (equal); funding acquisition (supporting); visualization (equal); writing – original draft (equal); writing – review and editing (equal). **Mark Burton:** Formal analysis (lead); methodology (equal); software (equal); visualization (equal); writing – review and editing (equal). **Kristina P. Sørensen:** Conceptualization (equal); data curation (equal); funding acquisition (equal); investigation (equal); writing – review and editing (equal). **Martin J. Larsen:** Investigation (equal); writing – review and editing (equal). **Thi T. N. Do:** Data curation (equal); visualization (equal); writing – review and editing (equal). **Martin Bak:** Data curation (equal); resources (equal); writing – review and editing (equal). **Søren Cold:** Data curation (equal); resources (equal); writing – review and editing (equal). **Mads Thomassen:** Conceptualization (equal); data curation (equal); methodology (equal); writing – review and editing (equal). **Qihua Tan:** Formal analysis (equal); methodology (equal); writing – review and editing (equal). **Torben A. Kruse:** Conceptualization (equal); funding acquisition (lead); supervision (lead); writing – review and editing (equal).

## FUNDING INFORMATION

This work was supported by the Danish Council for Independent Research (7016‐00346B/FSS, 09‐061677/FSS), the Danish Ministry of the Interior, the University of Southern Denmark (grant: DAWN2020), The Danish Council for Strategic Research (DBCG‐TIBCAT), Danish Cancer Society and Dansk Kræftforsknings Fond, Regionernes Medicin‐ og behandlingspulje, Breast Friends, Fonden til Lægevidenskabens Fremme, Meta & Håkon Baggers Fond, A. J. Andersen & Hustrus Fond, Inge & Jørgen Larsens Mindelegat, Overlægerådets Legatudvalg, Direktør Jacob Madsen & Hustru Olga Madsens Fond, Fru Ingeborg Albinus Larsens Mindelegat, Fonden af 1870, Harboefonden, Free Research Fond of the Odense University Hospital, Lundbeckfonden (Center of Excellence NanoCAN) and Frimodt‐Heineke Fonden.

## CONFLICT OF INTEREST STATEMENT

The authors declare no potential conflicts of interest.

## PRECIS

Our study shows that differential microRNA expression analysis has the potential to identify a subgroup of breast cancer patients with an extremely low long‐term risk of metastases. The ensemble‐based classification approach developed could form the starting point for a diagnostic tool to avoid unnecessary overtreatment of a significant proportion of breast cancer patients.

## Supporting information


Data S1.

## Data Availability

The data that support the findings of this study have been deposited in NCBI's Gene Expression Omnibus^17^ and are accessible through GEO Series accession numbers GSE126125 and GSE103161 (https://www.ncbi.nlm.nih.gov/geo/query/acc.cgi). Publicly available miRNA expression data published by D’Aiuto et al.^12^ were collected from NCBI's Gene Expression Omnibus database (accession number: GSE59829)^17^. Expression data published by Dvinge et al. and Curtis et al. (METABRIC)^11^, ^13^ were retrieved from the European Phenome‐Genome Archive (https://www.ebi.ac.uk/ega/home)^18^ after access was granted by METABRIC.

## References

[1] Ferlay J , Soerjomataram I , Dikshit R , et al. Cancer incidence and mortality worldwide: sources, methods and major patterns in GLOBOCAN 2012. Int J Cancer. 2015;136(5):E359‐E386. 25220842 10.1002/ijc.29210

[2] Bray F , Ferlay J , Soerjomataram I , Siegel RL , Torre LA , Jemal A . Global cancer statistics 2018: GLOBOCAN estimates of incidence and mortality worldwide for 36 cancers in 185 countries. CA Cancer J Clin. 2018;68(6):394‐424. 30207593 10.3322/caac.21492

[3] Mouridsen HT , Bjerre KD , Christiansen P , Jensen MB , Moller S . Improvement of prognosis in breast cancer in Denmark 1977–2006, based on the nationwide reporting to the DBCG registry. Acta Oncol. 2008;47(4):525‐536. 18465318 10.1080/02841860802027009

[4] Synnestvedt M , Borgen E , Russnes HG , et al. Combined analysis of vascular invasion, grade, HER2 and Ki67 expression identifies early breast cancer patients with questionable benefit of systemic adjuvant therapy. Acta Oncol. 2013;52(1):91‐101. 22934555 10.3109/0284186X.2012.713508

[5] Delahaye L , Drukker CA , Dreezen C , et al. A breast cancer gene signature for indolent disease. Breast Cancer Res Treat. 2017;164(2):461‐466. 28451965 10.1007/s10549-017-4262-0 PMC5487706

[6] Esserman LJ , Yau C , Thompson CK , et al. Use of molecular tools to identify patients with indolent breast cancers with ultralow risk over 2 decades. JAMA Oncol. 2017;3(11):1503‐1510. 28662222 10.1001/jamaoncol.2017.1261 PMC5710197

[7] Sjöström M , Chang SL , Fishbane N , et al. Comprehensive transcriptomic profiling identifies breast cancer patients who may be spared adjuvant systemic therapy. Clin Cancer Res. 2020;26(1):171‐182. 31558478 10.1158/1078-0432.CCR-19-1038

[8] Ohnstad HO , Borgen E , Falk RS , et al. Prognostic value of PAM50 and risk of recurrence score in patients with early‐stage breast cancer with long‐term follow‐up. Breast Cancer Res. 2017;19(1):120. 29137653 10.1186/s13058-017-0911-9 PMC5686844

[9] Kolbert CP , Feddersen RM , Rakhshan F , et al. Multi‐platform analysis of microRNA expression measurements in RNA from fresh frozen and FFPE tissues. PLoS One. 2013;8(1):e52517. 23382819 10.1371/journal.pone.0052517 PMC3561362

[10] van Schooneveld E , Wildiers H , Vergote I , Vermeulen PB , Dirix LY , Van Laere SJ . Dysregulation of microRNAs in breast cancer and their potential role as prognostic and predictive biomarkers in patient management. Breast Cancer Res. 2015;17:21. 25849621 10.1186/s13058-015-0526-y PMC4332424

[11] Curtis C , Shah SP , Chin SF , et al. The genomic and transcriptomic architecture of 2,000 breast tumours reveals novel subgroups. Nature. 2012;486(7403):346‐352. 22522925 10.1038/nature10983 PMC3440846

[12] D'Aiuto F , Callari M , Dugo M , et al. miR‐30e* is an independent subtype‐specific prognostic marker in breast cancer. Br J Cancer. 2015;113(2):290‐298. 26057454 10.1038/bjc.2015.206 PMC4506390

[13] Dvinge H , Git A , Graf S , et al. The shaping and functional consequences of the microRNA landscape in breast cancer. Nature. 2013;497(7449):378‐382. 23644459 10.1038/nature12108

[14] Moller S , Jensen MB , Ejlertsen B , et al. The clinical database and the treatment guidelines of the Danish Breast Cancer Cooperative Group (DBCG); its 30‐years experience and future promise. Acta Oncol. 2008;47(4):506‐524. 18465317 10.1080/02841860802059259

[15] Sørensen KP , Thomassen M , Tan Q , et al. Long non‐coding RNA expression profiles predict metastasis in lymph node‐negative breast cancer independently of traditional prognostic markers. Breast Cancer Res. 2015;17:55. 25887545 10.1186/s13058-015-0557-4 PMC4416310

[16] Block I , Burton M , Sorensen KP , et al. Association of miR‐548c‐5p, miR‐7‐5p, miR‐210‐3p, miR‐128‐3p with recurrence in systemically untreated breast cancer. Oncotarget. 2018;9(10):9030‐9042. 29507672 10.18632/oncotarget.24088 PMC5823652

[17] Edgar R , Domrachev M , Lash AE . Gene expression omnibus: NCBI gene expression and hybridization array data repository. Nucleic Acids Res. 2002;30(1):207‐210. 11752295 10.1093/nar/30.1.207 PMC99122

[18] Lappalainen I , Almeida‐King J , Kumanduri V , et al. The European Genome‐phenome Archive of human data consented for biomedical research. Nat Genet. 2015;47(7):692‐695. 26111507 10.1038/ng.3312 PMC5426533

[19] Larsen MJ , Thomassen M , Tan Q , Sorensen KP , Kruse TA . Microarray‐based RNA profiling of breast cancer: batch effect removal improves cross‐platform consistency. Biomed Res Int. 2014;2014:651751. 25101291 10.1155/2014/651751 PMC4101981

[20] Cruz JA , Wishart DS . Applications of machine learning in cancer prediction and prognosis. Cancer Informa. 2007;2:59‐77. PMC2675494 19458758

[21] Somorjai RL , Dolenko B , Baumgartner R . Class prediction and discovery using gene microarray and proteomics mass spectroscopy data: curses, caveats, cautions. Bioinformatics. 2003;19(12):1484‐1491. 12912828 10.1093/bioinformatics/btg182

[22] Burton M , Thomassen M , Tan Q , Kruse TA . Gene expression profiles for predicting metastasis in breast cancer: a cross‐study comparison of classification methods. TheScientificWorldJournal. 2012;2012:380495. 10.1100/2012/380495 PMC3515909 23251101

[23] Foekens JA , Sieuwerts AM , Smid M , et al. Four miRNAs associated with aggressiveness of lymph node‐negative, estrogen receptor‐positive human breast cancer. Proc Natl Acad Sci USA. 2008;105(35):13021‐13026. 18755890 10.1073/pnas.0803304105 PMC2529088

[24] Mestdagh P , Hartmann N , Baeriswyl L , et al. Evaluation of quantitative miRNA expression platforms in the microRNA quality control (miRQC) study. Nat Methods. 2014;11(8):809‐815. 24973947 10.1038/nmeth.3014

[25] Drukker CA , Schmidt MK , Rutgers EJ , et al. Mammographic screening detects low‐risk tumor biology breast cancers. Breast Cancer Res Treat. 2014;144(1):103‐111. 24469641 10.1007/s10549-013-2830-5 PMC3924026

[26] Esserman LJ , Thompson IM , Reid B , et al. Addressing overdiagnosis and overtreatment in cancer: a prescription for change. Research support, N.I.H., extramural. Lancet Oncol. 2014;15(6):e234‐e242. 24807866 10.1016/S1470-2045(13)70598-9 PMC4322920

[27] Candido Dos Reis FJ , Wishart GC , Dicks EM , et al. An updated PREDICT breast cancer prognostication and treatment benefit prediction model with independent validation. Breast Cancer Res. 2017;19(1):58. 28532503 10.1186/s13058-017-0852-3 PMC5440946

[28] Duffy MJ , Harbeck N , Nap M , et al. Clinical use of biomarkers in breast cancer: updated guidelines from the European group on tumor markers (EGTM). Eur J Cancer. 2017;75:284‐298. 28259011 10.1016/j.ejca.2017.01.017

[29] Krop I , Ismaila N , Andre F , et al. Use of biomarkers to guide decisions on adjuvant systemic therapy for women with early‐stage invasive breast cancer: American Society of Clinical Oncology Clinical Practice Guideline focused update. J Clin Oncol. 2017;35(24):2838‐2847. 28692382 10.1200/JCO.2017.74.0472 PMC5846188

[30] Lopes Cardozo JMN , Drukker CA , Rutgers EJT , et al. Outcome of patients with an ultralow‐risk 70‐gene signature in the MINDACT trial. J Clin Oncol. 2022;40(12):1335‐1345. 35061525 10.1200/JCO.21.02019

[31] Early Breast Cancer Trialists' Collaborative Group (EBCTCG) . Effects of chemotherapy and hormonal therapy for early breast cancer on recurrence and 15‐year survival: an overview of the randomised trials. Lancet. 2005;365(9472):1687‐1717. 15894097 10.1016/S0140-6736(05)66544-0

[32] Pan H , Gray R , Braybrooke J , et al. 20‐year risks of breast‐cancer recurrence after stopping endocrine therapy at 5 years. N Engl J Med. 2017;377(19):1836‐1846. 29117498 10.1056/NEJMoa1701830 PMC5734609

[33] van't Veer LJ , Dai H , van de Vijver MJ , et al. Gene expression profiling predicts clinical outcome of breast cancer. Nature. 2002;415(6871):530‐536. 11823860 10.1038/415530a

[34] van de Vijver MJ , He YD , van't Veer LJ , et al. A gene‐expression signature as a predictor of survival in breast cancer. N Engl J Med. 2002;347(25):1999‐2009. 12490681 10.1056/NEJMoa021967

[35] Damavandi Z , Torkashvand S , Vasei M , Soltani BM , Tavallaei M , Mowla SJ . Aberrant expression of breast development‐related micrornas, miR‐22, miR‐132, and miR‐212, breast tumor tissues. J Breast Cancer. 2016;19(2):148‐155. 27382390 10.4048/jbc.2016.19.2.148 PMC4929255

[36] Sathipati SY , Ho SY . Identifying a miRNA signature for predicting the stage of breast cancer. Sci Rep. 2018;8(1):16138. 30382159 10.1038/s41598-018-34604-3 PMC6208346

[37] Zhang H , Zhang Y , Yan W , et al. Association between three functional microRNA polymorphisms (miR‐499 rs3746444, miR‐196a rs11614913 and miR‐146a rs2910164) and breast cancer risk: a meta‐analysis. Oncotarget. 2017;8(1):393‐407. doi:10.18632/oncotarget.13426 27880723 PMC5352128

[38] Zhang K , Wang YW , Wang YY , et al. Identification of microRNA biomarkers in the blood of breast cancer patients based on microRNA profiling. Gene. 2017;619:10‐20. 28359916 10.1016/j.gene.2017.03.038

[39] Yu J , Zhang X , He R , et al. LINC01234 accelerates the progression of breast cancer via the miR‐525‐5p/cold shock domain‐containing E1 axis. Dis Markers. 2022;2022:6899777. 35923244 10.1155/2022/6899777 PMC9343190

[40] Tang C , Wang X , Ji C , et al. The role of miR‐640: a potential suppressor in breast cancer via Wnt7b/β‐catenin signaling pathway. Front Oncol. 2021;11:645682. 33912460 10.3389/fonc.2021.645682 PMC8072343

[41] Li Z , Zheng J , Lin W , et al. Circular RNA hsa_circ_0001785 inhibits the proliferation, migration and invasion of breast cancer cells in vitro and in vivo by sponging miR‐942 to upregulate SOCS3. Cell Cycle. 2020;19(21):2811‐2825. 33054543 10.1080/15384101.2020.1824717 PMC7714452

