# Supplementary material for: Ensemble‐based classification using microRNA expression identifies a breast cancer patient subgroup with an ultralow long‐term risk of metastases
Source: Cancer Med. 2024 Apr 27;13(9):e7089. doi: 10.1002/cam4.7089 (PMC11053369; doi:10.1002/cam4.7089)
Supplement: Supplementary file 1 — Data S1. [file CAM4-13-e7089-s001.docx]

*Supplement*

Ensemble-based classification using microRNA expression identifies a breast cancer patient subgroup with an ultralow long-term risk of metastases

Ines Block, PhD^1,2^; Mark Burton, PhD^1,3,4^; Kristina P. Sørensen, PhD^1^; Martin J. Larsen, PhD^1,3^; Thi T. N. Do, MsC^1,3^; Martin Bak, MD^5,6^; Søren Cold, MD^7^; Mads Thomassen, PhD^1,3,4^; Qihua Tan, PhD^3,4,8^; Torben A. Kruse, PhD^1,3,4*^

^1^ Department of Clinical Genetics, Odense University Hospital, Odense, Denmark

^2^ present address: Department of Mathematics & Computer Science, University of Marburg, Marburg, Germany

^3^ Human Genetics, Department of Clinical Research, University of Southern Denmark, Odense, Denmark

^4^ Clinical Genome Center, University of Southern Denmark & Region of Southern Denmark, Odense, Denmark

^5^ Department of Pathology, Odense University Hospital, Odense, Denmark

^6^ Department of Pathology, Hospital of Southwest Jutland, Esbjerg, Denmark

^7^ Department of Oncology, Odense University Hospital, Odense, Denmark

^8^ Epidemiology, Department of Public Health, University of Southern Denmark, Odense, Denmark

*Corresponding author

***Corresponding author***

Torben A. Kruse, PhD, Department of Clinical Genetics, Odense University Hospital, J.B. Winsløws Vej 4, 5000 Odense C, Denmark. Tel: +45-6541-1963. E-mail: [torben.kruse@rsyd.dk](mailto:torben.kruse@rsyd.dk)

**Supplementary Methods**

**Data Preprocessing**

Scanned images of the arrays were processed and quantified using the GenePixPro software. Raw intensity data were background corrected using the normexp method and bad quality flagged features were removed. Subsequently the background corrected data were normalized using lowess normalization within arrays and quantile normalization between arrays (R package limma).^1^ Log_2_ transformed ratios of the mean red intensity (Cy5) over mean green intensity (Cy3) was calculated for each probe, and replicate probes were collapsed by the median value. Missing values were imputed using k-nearest neighbors and the data were finally subjected to systematic bias correction by application of the ComBat function embedded in the *sva* R-package.

**Classification**

For classification seven different machine learning methods was applied encompassing: two support vector machines with a radial-based (RSVM) and linear-based kernel (LSVM), random forest (RF), Naïve Bayes (NB), Cox-risk score (COX-RS), k-nearest neighbors (KNN) and logistic regression (LR): The following classifications cutoffs were used for assignment of a sample to the metastatic class if: probability of recurrence ≥50% (RSVM, LSVM, RF, NB and LR), Cox-risk regression sum ≥ 0 (COX-RS) and class assignment of 2 (KNN). The optimal model during training was selected based on maximization of the mean of sensitivity and specificity, assessed using leave-one-pair-out-cross-validation (LOPOCV). Briefly, in this procedure a single pair of matched samples served as test samples and the remaining samples as a training set. This was repeated until all pairs had been left out once and the accuracy of the classifier was determined by the correctly classified samples. The LOPOCV procedure provides an unbiased performance estimate and is the optimal method in small datasets.^2,3^

In the training set, feature selection is necessary to avoid a small sample-per-feature ratio and has been shown to provide better classification.^3^ The feature selection procedure used in this study consisted of three steps: 1) testing the miRNA’s in the training set for significance of differential expression using the Student’s paired t-test; 2) Ranking the significant differentially expressed miRNA’s (FDR≤0.05) according to their random-forest importance value for a given feature, this value reports the standardized drop in prediction accuracy when the class labels are permuted ^4^ and 3) finding the optimal number of miRNAs - by subsequently adding one miRNA at a time in a top-down forward-wrapper approach starting with the top two miRNAs of the ranked list; at each increment the classification accuracy of the training samples was assessed using LOPOCV in a nested inner loop^5^. Fisher’s exact test was used to calculate the significance of the classification results. The pipeline for this classification procedure is illustrated in Supplementary Figure S1A.

**Voting Procedure**

For each sample the voting classification procedure aggregated the classification decisions from the seven individual machine learning methods into a count of recurrence assignments, thus giving a range from 0 to 7 votes for recurrence assignments. In our OUH training dataset and the METABRIC validation set, samples receiving zero votes indicate an ultralow risk for recurrence and those samples achieving 0 or 1 votes indicate low risk for recurrence. Due to the voting distribution in the D’Aiuto *et al.* data set a modified threshold for risk group assignment was applied, whereby samples receiving two votes were considered as ultralow risk and samples receiving two and three votes were considered as low risk. The pipeline illustrating the voting procedure is presented in Figure 2.

**Optimal miRNA signatures**

The above-mentioned classification resulted in the same number of different models and of miRNA sets as the number of pairs, which is unsuitable for validation in independent datasets. To obtain optimal miRNA profiles for validation purposes, we used the entire dataset for feature selection as described above in Supplementary Figure S1B. We evaluated the performance of the optimal classifier using LOPOCV by adding one feature at a time in a top-down selection starting with the top two features of the ranked miRNAs.

**Validation in independent datasets**

To examine the performance of the miRNA profiles developed from the OUH data, we identified two miRNA expression datasets (GSE59829 by D’Aiuto *et al*.^6^ and the METABRIC expression data published by Dvinge *et al.*^7^ and Curtis *et al.*^8^), which were suitable for validation. For consistent miRNA nomenclature the R-package *miRNAmeConverter* was applied and MIMAT numbers were used as identifiers in subsequent analysis.^9^ The lists of miRNAs selected by OUH were used for model building and classification of samples using Leave-one-pair-out-cross-validation within the two validation datasets. A number of miRNAs from the OUH derived profiles were missing in the two validation sets as only 859 or 815 mature miRNAs were analyzed in the D’Aiuto *et al.* and METABRIC studies, respectively. Therefore, validation was restricted using these reduced lists of miRNAs.

**Statistical Analysis**

Classification significance was calculated using a one-tailed Fisher’s exact test. The 95% confidence intervals (CI) for the classification accuracies determined by leave-one-out cross validation in the validation sets was performed using the Clopper-Pearson method^10,11^ and the binom.test function embedded in the stats R-package. Kaplan-Meier survival plots demonstrated differential recurrence-free survival in the predicted subgroups of samples. The associated significance and hazard ratios (HR) with a 95%CI were determined using the Cox proportional hazards regression model. For this purpose, the *survival* R-package was used. Logistic regression analysis was performed to examine the independence of established clinical markers such as age at diagnosis, tumor size and grade on the classification predictions and considered significant if the P-value was ≤0.05.

**Code availability**

All calculations were performed using the open source R-environment (version 3.4.4; https://www.R-project.org)^12^ and the following R-packages: *stats*, *e1071* (https://CRAN.R-project.org/package=e1071), *randomForest* (https://CRAN.R-project.org/package=randomForest), *survival* (https://cran.r-project.org/web/package=survival), *class* (https://CRAN.R-project.org/package=class), *limma*^13^ and *miRNAmeConverter*^9^*.* The latter are available from Bioconductor at <https://bioconductor.org/packages/miRNAmeConverter/> and <https://bioconductor.org/packages/limma/>.

**Supplementary Figures**


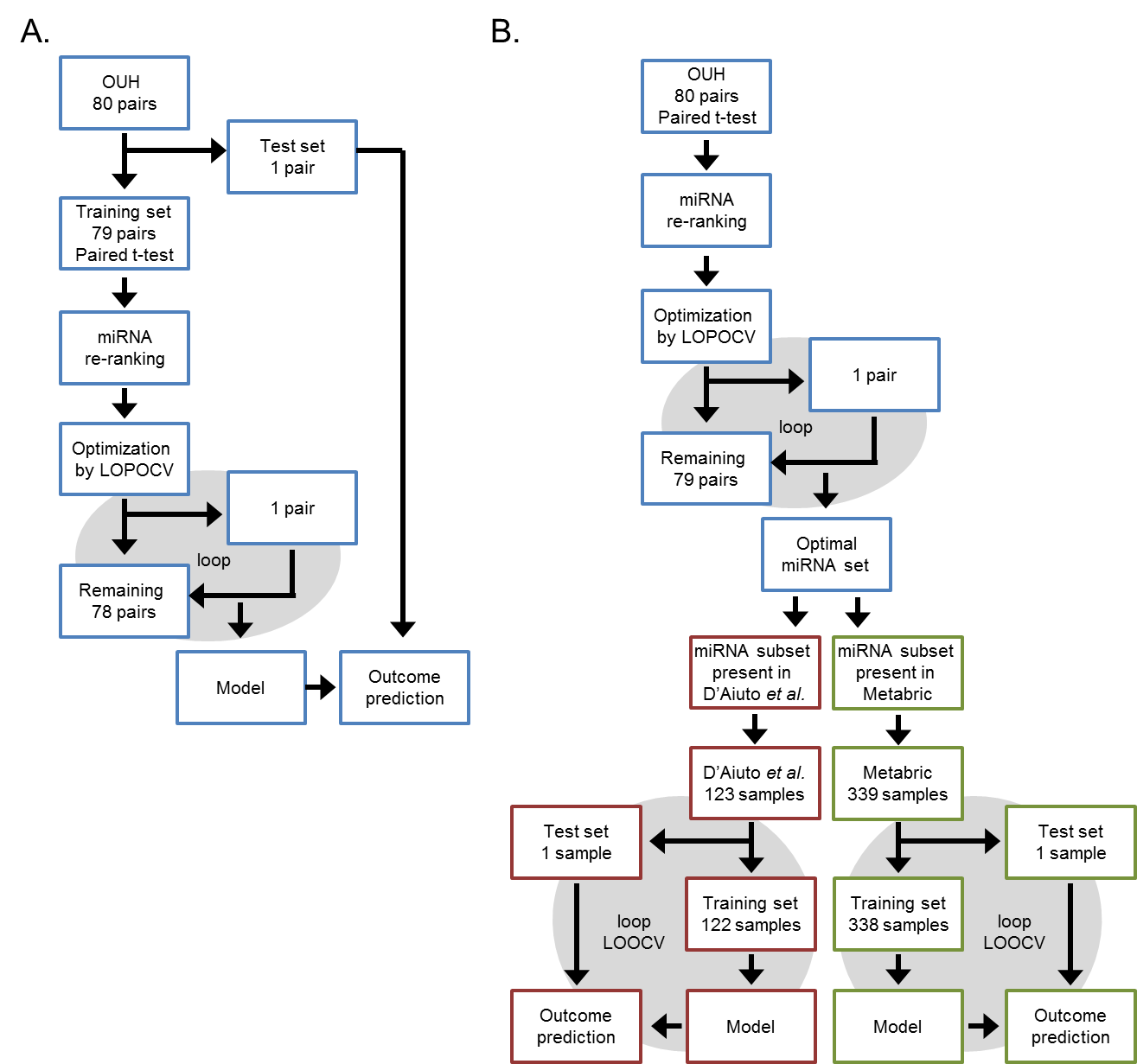


**Figure S1. Leave-one-out model building and outcome prediction**

(A) Outcome prediction in the OUH cohort by leave-one-pair-out-cross-validation (LOPOCV). One pair was defined as test sample while the remaining 79 pairs were used for training. Significantly differentially expressed miRNAs between patients with and without recurrence were identified using a paired Student’s t-test, and this subset was re-ranked accordingly to their Random Forest variable importance measure (RF-VIM). Starting with the highest ranked miRNAs each of the seven machine learning methods identified the optimal miRNA set by LOPOCV to predict outcome in one selected pair using 78 pairs for training (inner loop, grey). The procedure was repeated until each pair had been left out once. The finally optimal miRNA set/model was applied to predict outcome in the initially left out test pair. The entire procedure was repeated until all pairs had been left out as a test pair and their outcome predicted independently of the inner training loop. (B) Significantly differentially expressed miRNAs between patients with and without recurrence in all 80 pairs were identified using a paired Student’s t-test. Subsequently, these miRNAs were again re-ranked by the RF-VIM. Starting with the highest ranked miRNAs each of the seven machine learning methods identified an optimal miRNA set via LOPOCV (inner loop, grey) for subsequent validation in the independent datasets. Those miRNAs present in the D’Aiuto *et al.* (red encased) or METABRIC (green encased) dataset were used for outcome prediction via leave-one-out-cross-validation (LOOCV). Again, each of the seven machine learning methods identified an optimal model to predict outcome in one left out sample while using the remaining samples for training.

**Figure S2. Overall classification using (A) RSVM, (B) RF, (C) NB, (D) LSVM, (E) COX-RS, (F) KNN, (G) LR.** Dot plots illustrate the probability of recurrence versus the actual clinical outcome and ER status of the patients

**Figure S3. Kaplan-Meier survival curves of recurrence-free survival for (A) estrogen receptor positive and (B) estrogen receptor negative patients classified as ultralow (left) or low-risk (right) patients using voting.** Hazard ratios (HR) including 95 percent confidence intervals are shown in parenthesis if applicable. Significance of differences in recurrence-free survival was tested using a Cox-proportional hazards regression model (COX-PH). n/a: not applicable

**Figure S4. Outcome prediction based on voting and Kaplan-Meier curves in the (A) D’Aiuto *et al.* and (B) METABRIC data set.** Voting results for all estrogen receptor positive (ERpos) and negative (ERneg) patients are displayed as dot plots. For the D’Aiuto *et al.* data set only prediction results of only 6 methods were considered as the COX risk score could not be determined. Vertical lines discriminate metastatic and recurrence-free patients while horizontal lines indicate differences between predicted low-risk and high-risk patients. Hazard ratios (HR) for survival analysis including 95 percent confidence intervals are shown if applicable. Significance of differences in recurrence-free and overall survival was tested using a Cox-proportional hazards regression model (COX-PH).n/a: not applicable

**Supplementary Tables**

**Table S1. Risk factors and selection criteria for low-risk breast cancer patients who did not receive any systemic adjuvant treatment from 1980-2003 as recommended by the Danish Breast Cancer Cooperative Group (DBCG).** Data extracted from Møller *et al.*^14^

|  | **DBCG 82** | **DBCG 89** | **DBCG 99** | **DBCG 01** |
| --- | --- | --- | --- | --- |
| **Risk factor** |  | | | |
| Age | ≤ 70 years | ≤ 75 years | ≤ 70 years | ≥ 35 years |
| Lymph node status | negative | negative | negative | negative |
| Tumour size | ≤ 5 cm | ≤ 5 cm | ≤ 5 cm | ≤ 2 cm |
| Histology and grade |  | Grade I if ductal^1^ | Grade I if ductal | Grade I if ductal |
| ER and PgR status |  |  | Positive or unknown | Positive or unknown |
| ^1^ Pre-menopausal only | | | | |

**Table S2. Overall classification using a 50% probability of recurrence cut off (RSVM, RF, NB, LSVM and LR), Regression sum of >=0 (COX-RS) or class membership (KNN), to discern patients with low and high risk for recurrence.**

| **Method** | **Sensitivity** | **Specificity** | **Accuracy^a^** | ***P*^b^** |
| --- | --- | --- | --- | --- |
| Radial-based kernel Support Vector Machine (RSVM) | 81 | 84 | 82 | 1.1x10^-16^ |
| Random Forest (RF) | 84 | 88 | 86 | 9.84x10^-22^ |
| Naïve Bayes (NB) | 88 | 80 | 84 | 9.92x10^-19^ |
| Linear kernel Support Vector Machine (LSVM) | 82 | 86 | 84 | 9.92x10^-19^ |
| COX risk score (COX RS) | 76 | 78 | 77 | 4.96x10^-12^ |
| K-Nearest Neighbor (KNN) | 82 | 86 | 84 | 9.92x10^-19^ |
| Logistic Regression (LR) | 81 | 86 | 84 | 9.92x10^-19^ |

^a^Mean of sensitivity and specificity; ^b^*P*-value as determined via Fisher’s exact test, one-tailed

**Table S3. Current criteria for treatment selection according to the recommendations of the Danish Breast Cancer Cooperative Group (DBCG).** Patients belonging to DBCG risk group I may not receive any adjuvant systemic treatment while patients belonging to DBCG risk group II receive treatment as detailed in Supplementary Table A4.

| **Age (years)** | **Tumor size (mm)** | **No. of positive LN** | **Type and grade** | **ER status (%pos)** | **ERBB2 status** | **DBCG group** |
| --- | --- | --- | --- | --- | --- | --- |
| ≥60 | ≤10 | 0 | IDC I, ILC I-II, other types | ≥10 or n/a modular^a^ | Negativ/na | I |
|  |  |  |  |  | Positive | II |
|  |  |  |  | 0-9% | n/r | II |
|  |  |  | IDC II-III, ILC III | n/r | n/r | II |
|  |  | ≥1 | n/r | n/r | n/r | II |
|  | >10 | n/r | n/r | n/r | n/r | II |
| <60 | n/r | n/r | n/r | n/r | n/r | II |
| LN: lymph node, IDC: Invasive Ductual Carcinoma, ILC: Invasive Lobular Carcinoma, n/a: unknown, n/r: not relevant; ^a^modular or modular negative, immunohistochemistry | | | | | | |

**Table S4. Treatment selection according to the recommendations of the Danish Breast Cancer Cooperative Group (DBCG)**

| **DBCG group** | **ERBB2 status** | **ER status^a^ (%pos)** | | **Age (years)** | **Menopause** | **Risk-factors^b^** | **Treatment** |
| --- | --- | --- | --- | --- | --- | --- | --- |
| I | n/r | n/r | | n/r | n/r | n/r | None |
| II | Positive | 0 | | n/r | n/r | n/r | Chemo + ERBB2 |
|  |  | ≥1 | | n/r | n/r | n/r | Chemo + ERBB2 + Endo |
|  | Negative | 0 | | n/r | n/r | n/r | Chemo |
|  |  | 1-9 | | n/r | n/r | n/r | Chemo + Endo |
|  |  | ≥10 | | <40 | n/r | n/r | Chemo + Endo |
|  |  |  |  | 40-49 | n/r | No | Endo |
|  |  |  |  |  | n/r | Yes | Chemo + Endo |
|  |  |  |  | ≥50 | pre | No | Endo |
|  |  |  |  |  |  | Yes | Chemo + Endo |
|  |  |  |  |  | post | No | Endo |
|  |  |  |  |  |  | Yes | Chemo + Endo |
| ^a^Immunohistochemistry; ^b^Risk factors: Patients ≥40 years in age with ERBB2 negative and estrogen receptor (ER) positive (≥10%) tumors will be offered chemotherapy in case one of the risk factors listed below is fulfilled | | | | | | | |
| 40-49 years | | | Yes: Size≥10 mm or LN positive or IDC II-III or ILC III | | | | |
| Premenopausal, ≥ 50 years | | | Yes: Size≥20 mm or LN positive or IDC II-III or ILC III | | | | |
| Postmenopausal, ≥ 50 years | | | Yes: DBCG score Q2-Q4 or ≥4 positive LNs | | | | |
| Chemo: Chemotherapy, ERBB2: ERBB2 targeted therapy, Endo: estrogen receptor targeted therapy, LN: lymph node, %pos: Percent of positive cells | | | | | | | |

**Table S5. Overview of prediction performance for estrogen receptor (ER) positive and negative patients^a^ using voting.**

| **Data set** | **Total no. of patients** | **Patients wo rec correctly classified as ultralow** | **Patients w rec incorrectly**  **classified as ultralow risk** | ***P*-value^b^** | **Patients wo rec correctly classified as low risk** | **Patients w rec incorrectly**  **classified as low risk** | ***P*-value^b^** |
| --- | --- | --- | --- | --- | --- | --- | --- |
|  |  | Ultralow risk | | | Low risk | | |
| ER positive^a^ | | | | | | | |
| OUH | 120 | 29/62 (46.8%) | 0/58 (0.0%) | 7.32x10^-11^ | 32/62 (53.2%) | 4/58 (6.9%) | 3.77x10^-8^ |
| D’Aiuto *et al.*^c^ | 100 | 12/53 (22.6%) | 0/47 (0.0%) | 0.00025 | 41/53 (77.4%) | 12/47 (25.5%) | 1.82x10^-7^ |
| META-BRIC | 249 | 1/184 (0.54%) | 1/65 (1.54%) | 0.93 | 28/184 (15.2%) | 2/65 (3.08%) | 0.0051 |
| ER negative^a^ | | | | | | | |
| OUH | 40 | 8/18 (44.4%) | 0/22 (0.0%) | 0.00057 | 14/18 (77.8%) | 0/22 (0.0%) | 1.32x10^-7^ |
| D’Aiuto *et al.*^c^ | 23 | 4/11 (36.4%) | 0/12 (0.0%) | 0.037 | 9/11 (81.8%) | 2/12 (16.7%) | 0.0028 |
| META-BRIC | 90 | 2/77 (2.60%) | 0/13 (0.0%) | 0.73 | 7/77 (9.09%) | 1/13 (7.69%) | 0.67 |
| wo rec: without recurrence; w rec: with recurrence; ^a^defined by mRNA expression; ^b^*P*-value indicating statistical significance as determined via Fisher’s exact test, one-tailed; ^c^The ultralow risk group was defined as patients receiving 2 votes and the low risk group as patients receiving 2 or 3 votes as no patient received 0 or 1 votes (see also Supplementary Figure S4) | | | | | | | |

**Table S6. Selection of an optimal miRNA set for outcome prediction in the OUH training set and subsequent validation in METABRIC^7,8^ and D’Aiuto *et al*.^6^ data sets.** A reduced number of miRNAs (miRs red.) was used for outcome prediction in the validation sets because not all miRNAs were *a priori* analyzed. Seven different classification methods were applied.

|  | **OUH - Training** | | **METABRIC - Validation** | | | | | | **D’Aiuto *et al.* - Validation** | | | | | |
| --- | --- | --- | --- | --- | --- | --- | --- | --- | --- | --- | --- | --- | --- | --- |
| Method | No. of miRs | Acc^a^ | miRs red. | Sens | Spec | Acc^a^ | *P*^b^ | 95% CI^b^ | miRs  red. | Sens | Spec | Acc^a^ | *P*^b^ | 95% CI^b^ |
| RSVM | 30 | 90 | 12 | 62 | 48 | 55 | 0.11 | 47.0-63.0 | 21 | 83 | 94 | 88 | 1.81x10^-18^ | 80.9-93.4 |
| RF | 107 | 92.5 | 42 | 55 | 57 | 56 | 0.087 | 47.6-63.7 | 69 | 80 | 34 | 57 | 0.084 | 47.3-65.9 |
| NB | 38 | 90 | 15 | 60 | 61 | 61 | 0.0040 | 52.8-68.6 | 26 | 3 | 98 | 51 | 0.46 | 41.5-60.2 |
| LSVM | 29 | 89.4 | 11 | 99 | 5 | 52 | 0.34 | 43.8-60.0 | 20 | 8 | 95 | 52 | 0.39 | 42.3-61.0 |
| COX RS | 109 | 95 | 37 | 62 | 55 | 58 | 0.033 | 49.5-65.6 | 69 | n/a | n/a | n/a | n/a | n/a |
| LR | 10 | 87.3 | 4 | 58 | 55 | 56 | 0.087 | 47.6-63.7 | 10 | 100 | 6 | 53 | 0.26 | 44.0-62.6 |
| KNN | 10 | 88 | 6 | 32 | 75 | 53 | 0.24 | 45.1-61.2 | 10 | 39 | 70 | 55 | 0.16 | 45.7-64.3 |

^a^training accuracy (OUH Acc) and balanced accuracy (Acc) in validation sets calculated as mean of Sensitivity (Sens) and specificity (Spec); ^b^*P*-value indicating significance of prediction as determined via Clopper-Pearson method^11^; CI: confidence interval; n/a: not applicable/available; RSVM: Radial-based kernel Support Vector Machine; RF: Random Forest; NB: Naïve Bayes; LSVM: Linear kernel Support Vector Machine; COX-RS: COX risk score; KNN: K-Nearest Neighbor; LR: Logistic Regression

**Table S7. Overview of miRs used for outcome prediction by Radial-based kernel Support Vector Machine (RSVM), Random Forest (RF), Naïve Bayes (NB), Linear Support Vector Machine (LSVM), COX risk score (COX RS), K-Nearest Neighbor (KNN) and Logistic Regression (LR) in the OUH training set (O) and validation in the METABRIC (M) and D’Aiuto *et al.* (D) data sets.**

| **MIMAT** | **miR name** | **R-SVM** | | | **RF** | | | **NB** | | | **LSVM** | | | **COX RS** | | | **LR** | | | **KNN** | | | **common**  **OUH** | **common**  **all cohorts** |
| --- | --- | --- | --- | --- | --- | --- | --- | --- | --- | --- | --- | --- | --- | --- | --- | --- | --- | --- | --- | --- | --- | --- | --- | --- |
|  |  | **O** | **M** | **D** | **O** | **M** | **D** | **O** | **M** | **D** | **O** | **M** | **D** | **O** | **M** | **D** | **O** | **M** | **D** | **O** | **M** | **D** |  |  |
|  |  | **30** | **12** | **21** | **107** | **42** | **68** | **38** | **15** | **26** | **29** | **11** | **20** | **109** | **37** | **65** | **10** | **4** | **10** | **10** | **6** | **10** |  |  |
| MIMAT0000269 | hsa-miR-212-3p | x | x | x | x | x | x | x | x | x | x | x | x | x | x | x | x | x | x | x | x | x | 7 | 21 |
| MIMAT0002870 | hsa-miR-499-5p | x | x | x | x | x | x | x | x | x | x | x | x | x | x | x | x | x | x | x | x | x | 7 | 21 |
| MIMAT0002828 | hsa-miR-519e-5p | x | x | x | x | x | x | x | x | x | x | x | x | x | x | x | x |  | x | x | x | x | 7 | 20 |
| MIMAT0002838 | hsa-miR-525-5p | x | x | x | x | x | x | x | x | x | x | x | x | x | x | x | x | x | x | x | x | x | 7 | 21 |
| MIMAT0003310 | hsa-miR-640 | x |  | x | x |  | x | x |  | x | x |  | x | x |  | x | x |  | x | x |  | x | 7 | 14 |
| MIMAT0004985 | hsa-miR-942-5p | x |  | x | x |  | x | x |  | x | x |  | x | x |  | x | x |  | x | x |  | x | 7 | 14 |
| MIMAT0004608 | hsa-miR-146a-3p | x |  | x | x |  | x | x |  | x | x |  | x | x |  | x |  |  |  | x |  | x | 6 | 12 |
| MIMAT0003283 | hsa-miR-615-3p | x | x | x | x | x | x | x | x | x | x | x | x | x | x | x |  |  |  | x |  | x | 6 | 17 |
| MIMAT0002808 | hsa-miR-511-5p | x |  | x | x |  | x | x |  | x | x |  | x | x |  | x | x |  | x |  |  |  | 6 | 12 |
| MIMAT0003234 | hsa-miR-569 | x |  | x | x |  | x | x |  | x | x |  | x | x |  | x | x |  | x |  |  |  | 6 | 12 |
| MIMAT0003241 | hsa-miR-576-5p | x |  | x | x |  | x | x |  | x | x |  | x | x |  | x | x |  | x |  |  |  | 6 | 12 |
| MIMAT0005589 | hsa-miR-1234-3p | x |  | x | x |  | x | x |  | x |  |  |  | x |  | x |  |  |  | x |  | x | 6 | 10 |
| MIMAT0005921 | hsa-miR-1267 | x |  | x | x | x | x | x | x | x | x |  | x | x | x | x |  |  |  |  |  |  | 5 | 13 |
| MIMAT0005887 | hsa-miR-1299 | x | x | x | x | x | x | x | x | x | x | x | x | x | x | x |  |  |  |  | x |  | 5 | 16 |
| MIMAT0004589 | hsa-miR-30b-3p | x |  | x | x | x | x | x | x | x | x |  | x | x | x | x |  |  |  |  |  |  | 5 | 13 |
| MIMAT0015063 | hsa-miR-3183 | x |  |  | x |  |  | x |  |  | x |  |  | x |  |  |  |  |  |  |  |  | 5 | 5 |
| MIMAT0017392 | hsa-miR-3200-5p | x |  |  | x |  |  | x |  |  | x |  |  | x |  |  |  |  |  |  |  |  | 5 | 5 |
| MIMAT0018082 | hsa-miR-3661 | x |  |  | x |  |  | x |  |  | x |  |  | x |  |  |  |  |  |  |  |  | 5 | 5 |
| MIMAT0016924 | hsa-miR-4330 | x |  |  | x |  |  | x |  |  | x |  |  | x |  |  |  |  |  |  |  |  | 5 | 5 |
| MIMAT0004777 | hsa-miR-513a-3p | x |  | x | x |  | x | x |  | x | x |  | x | x |  | x |  |  |  |  |  |  | 5 | 10 |
| MIMAT0002827 | hsa-miR-515-3p | x | x | x | x | x | x | x | x | x | x |  | x | x | x | x |  |  |  |  | x |  | 5 | 15 |
| MIMAT0002834 | hsa-miR-520a-3p | x |  | x | x |  | x | x |  | x | x |  | x | x |  | x |  |  |  |  |  |  | 5 | 10 |
| MIMAT0005889 | hsa-miR-548l | x |  | x | x |  | x | x |  | x | x |  | x | x |  | x |  |  |  |  |  |  | 5 | 10 |
| MIMAT0015060 | hsa-miR-548w | x |  |  | x |  |  | x |  |  | x |  |  | x |  |  |  |  |  |  |  |  | 5 | 5 |
| MIMAT0003257 | hsa-miR-550a-3p | x |  |  | x | x |  | x | x |  | x |  |  | x | x |  |  |  |  |  |  |  | 5 | 8 |
| MIMAT0004916 | hsa-miR-888-5p | x |  | x | x |  | x | x |  | x | x |  | x | x |  | x |  |  |  |  |  |  | 5 | 10 |
| n/a | hsa-miRPlus-I320a* | x |  |  | x |  |  | x |  |  | x |  |  | x |  |  |  |  |  |  |  |  | 5 | 5 |
| n/a | hsa-miRPlus-J212* | x |  |  | x |  |  | x |  |  | x |  |  | x |  |  |  |  |  |  |  |  | 5 | 5 |
| MIMAT0004672 | hsa-miR-106b-3p | x | x | x | x | x | x | x | x | x | x | x | x |  |  |  |  |  |  |  |  |  | 4 | 12 |
| MIMAT0017981 | hsa-miR-3605-5p | x |  |  | x |  |  | x |  |  | x |  |  |  |  |  |  |  |  |  |  |  | 4 | 4 |
| MIMAT0004502 | hsa-miR-28-3p |  |  |  | x | x | x | x | x | x |  |  |  |  |  |  |  |  |  | x |  | x | 3 | 8 |

**Table S7. continued**

| **MIMAT** | **miR name** | **R-SVM** | | | **RF** | | | **NB** | | | **LSVM** | | | **COX RS** | | | **LR** | | | **KNN** | | | **common**  **OUH** | **common**  **all cohorts** |
| --- | --- | --- | --- | --- | --- | --- | --- | --- | --- | --- | --- | --- | --- | --- | --- | --- | --- | --- | --- | --- | --- | --- | --- | --- |
|  |  | **O** | **M** | **D** | **O** | **M** | **D** | **O** | **M** | **D** | **O** | **M** | **D** | **O** | **M** | **D** | **O** | **M** | **D** | **O** | **M** | **D** |  |  |
|  |  | **30** | **12** | **21** | **107** | **42** | **68** | **38** | **15** | **26** | **29** | **11** | **20** | **109** | **37** | **65** | **10** | **4** | **10** | **10** | **6** | **10** |  |  |
| MIMAT0005877 | hsa-miR-1286 |  |  |  | x |  | x | x |  |  |  |  |  | x |  | x |  |  |  |  |  |  | 3 | 5 |
| MIMAT0018083 | hsa-miR-3662 |  |  |  | x |  |  | x |  |  |  |  |  | x |  |  |  |  |  |  |  |  | 3 | 3 |
| MIMAT0003221 | hsa-miR-557 |  |  |  | x | x | x | x | x | x |  |  |  | x | x | x |  |  |  |  |  |  | 3 | 9 |
| MIMAT0003223 | hsa-miR-559 |  |  |  | x |  | x | x |  | x |  |  |  | x |  | x |  |  |  |  |  |  | 3 | 6 |
| n/a | hsa_SNORD15A |  |  |  | x |  |  | x |  |  |  |  |  | x |  |  |  |  |  |  |  |  | 3 | 3 |
| n/a | hsa-miRPlus-I152* |  |  |  | x |  |  | x |  |  |  |  |  | x |  |  |  |  |  |  |  |  | 3 | 3 |
| MIMAT0000251 | hsa-miR-147a |  |  |  | x |  | x |  |  |  |  |  |  | x |  | x |  |  |  |  |  |  | 2 | 3 |
| MIMAT0004928 | hsa-miR-147b-3p |  |  |  | x | x | x | x | x | x |  |  |  |  |  |  |  |  |  |  |  |  | 2 | 6 |
| MIMAT0004604 | hsa-miR-127-5p |  |  |  | x |  | x |  |  |  |  |  |  |  |  |  | x |  | x |  |  |  | 2 | 4 |
| MIMAT0005955 | hsa-miR-1197 |  |  |  | x |  | x |  |  |  |  |  |  | x |  | x |  |  |  |  |  |  | 2 | 4 |
| MIMAT0000421 | hsa-miR-122-5p |  |  |  | x |  | x |  |  |  |  |  |  | x |  | x |  |  |  |  |  |  | 2 | 4 |
| MIMAT0004591 | hsa-miR-124-5p |  |  |  | x |  | x |  |  |  |  |  |  | x |  | x |  |  |  |  |  |  | 2 | 4 |
| MIMAT0005899 | hsa-miR-1247-5p |  |  |  | x | x | x |  |  |  |  |  |  | x | x | x |  |  |  |  |  |  | 2 | 6 |
| MIMAT0005799 | hsa-miR-1283 |  |  |  | x |  | x |  |  |  |  |  |  | x |  | x |  |  |  |  |  |  | 2 | 4 |
| MIMAT0004606 | hsa-miR-136-3p |  |  |  | x | x | x |  |  |  |  |  |  | x | x | x |  |  |  |  |  |  | 2 | 6 |
| MIMAT0007892 | hsa-miR-1915-3p |  |  |  | x |  |  |  |  |  |  |  |  | x |  |  |  |  |  |  |  |  | 2 | 2 |
| MIMAT0004491 | hsa-miR-19b-1-5p |  |  |  | x | x | x |  |  |  |  |  |  | x | x | x |  |  |  |  |  |  | 2 | 6 |
| MIMAT0014982 | hsa-miR-3120-3p |  |  |  | x |  |  |  |  |  |  |  |  | x |  |  |  |  |  |  |  |  | 2 | 2 |
| MIMAT0015043 | hsa-miR-3168 |  |  |  | x |  |  |  |  |  |  |  |  | x |  |  |  |  |  |  |  |  | 2 | 2 |
| MIMAT0004506 | hsa-miR-33a-3p |  |  |  | x |  | x |  |  |  |  |  |  | x |  | x |  |  |  |  |  |  | 2 | 4 |
| MIMAT0018081 | hsa-miR-3660 |  |  |  | x |  |  |  |  |  |  |  |  | x |  |  |  |  |  |  |  |  | 2 | 2 |
| MIMAT0018090 | hsa-miR-3667-3p |  |  |  | x |  |  |  |  |  |  |  |  | x |  |  |  |  |  |  |  |  | 2 | 2 |
| MIMAT0001621 | hsa-miR-369-5p |  |  |  | x | x | x |  |  | x |  |  |  | x | x | x |  |  |  |  |  |  | 2 | 7 |
| MIMAT0002172 | hsa-miR-376b-3p |  |  |  | x | x | x |  |  |  |  |  |  | x | x | x |  |  |  |  |  |  | 2 | 6 |
| MIMAT0018196 | hsa-miR-3921 |  |  |  | x |  |  |  |  |  |  |  |  | x |  |  |  |  |  |  |  |  | 2 | 2 |
| MIMAT0018350 | hsa-miR-3935 |  |  |  | x |  |  |  |  |  |  |  |  | x |  |  |  |  |  |  |  |  | 2 | 2 |
| MIMAT0018360 | hsa-miR-3944-3p |  |  |  | x |  |  |  |  |  |  |  |  | x |  |  |  |  |  |  |  |  | 2 | 2 |
| MIMAT0001343 | hsa-miR-425-3p |  | x |  | x | x | x |  |  |  |  |  |  | x | x | x |  |  |  |  |  |  | 2 | 7 |
| MIMAT0016899 | hsa-miR-4264 |  |  |  | x |  |  |  |  |  |  |  |  | x |  |  |  |  |  |  |  |  | 2 | 2 |
| MIMAT0016897 | hsa-miR-4269 |  |  |  | x | x |  |  |  |  |  |  |  | x | x |  |  |  |  |  |  |  | 2 | 4 |
| MIMAT0001635 | hsa-miR-452-5p |  |  |  | x | x | x |  |  |  |  |  |  | x | x | x |  |  |  |  |  |  | 2 | 6 |

**Table S7. continued**

| **MIMAT** | **miR name** | **R-SVM** | | | **RF** | | | **NB** | | | **LSVM** | | | **COX RS** | | | **LR** | | | **KNN** | | | **common**  **OUH** | **common**  **all cohorts** |
| --- | --- | --- | --- | --- | --- | --- | --- | --- | --- | --- | --- | --- | --- | --- | --- | --- | --- | --- | --- | --- | --- | --- | --- | --- |
|  |  | **O** | **M** | **D** | **O** | **M** | **D** | **O** | **M** | **D** | **O** | **M** | **D** | **O** | **M** | **D** | **O** | **M** | **D** | **O** | **M** | **D** |  |  |
|  |  | **30** | **12** | **21** | **107** | **42** | **68** | **38** | **15** | **26** | **29** | **11** | **20** | **109** | **37** | **65** | **10** | **4** | **10** | **10** | **6** | **10** |  |  |
| MIMAT0003161 | hsa-miR-493-3p |  |  |  | x | x | x |  |  |  |  |  |  | x | x | x |  |  |  |  |  |  | 2 | 6 |
| MIMAT0004975 | hsa-miR-509-3-5p |  |  |  | x |  | x |  |  |  |  |  |  | x |  | x |  |  |  |  |  |  | 2 | 4 |
| MIMAT0002852 | hsa-miR-517a-3p |  |  |  | x | x | x |  |  |  |  |  |  | x | x | x |  |  |  |  |  |  | 2 | 6 |
| MIMAT0002858 | hsa-miR-520g-3p |  |  |  | x | x | x |  | x |  |  |  |  | x | x | x |  |  |  |  |  |  | 2 | 7 |
| MIMAT0002867 | hsa-miR-520h |  |  |  |  |  | x |  |  |  |  |  |  |  |  | x |  |  |  |  |  |  | 0 | 2 |
| MIMAT0004954 | hsa-miR-543 |  |  |  | x | x | x |  |  |  |  | x |  | x | x | x |  |  |  |  |  |  | 2 | 7 |
| MIMAT0003323 | hsa-miR-548d-3p |  |  |  | x |  | x |  |  |  |  |  |  | x |  | x |  |  |  |  |  |  | 2 | 4 |
| MIMAT0003272 | hsa-miR-604 |  |  |  | x |  | x |  |  |  |  |  |  | x |  | x |  |  |  |  |  |  | 2 | 4 |
| MIMAT0004807 | hsa-miR-624-3p |  |  |  | x | x | x |  |  |  |  |  |  | x | x | x |  |  |  |  |  |  | 2 | 6 |
| MIMAT0004810 | hsa-miR-629-5p |  |  |  | x | x | x |  |  |  |  |  |  | x | x | x |  |  |  |  |  |  | 2 | 6 |
| MIMAT0005949 | hsa-miR-664-3p |  | x |  | x | x | x |  |  |  |  | x |  | x | x | x |  |  |  |  |  |  | 2 | 8 |
| MIMAT0004510 | hsa-miR-96-3p |  |  |  | x |  | x |  |  |  |  |  |  | x |  | x |  |  |  |  |  |  | 2 | 4 |
| n/a | hsa-miRPlus-A1072 |  |  |  | x |  |  |  |  |  |  |  |  | x |  |  |  |  |  |  |  |  | 2 | 2 |
| n/a | hsa-miRPlus-C1070 |  |  |  | x |  |  |  |  |  |  |  |  | x |  |  |  |  |  |  |  |  | 2 | 2 |
| MIMAT0005944 | hsa-miR-1252-5p |  |  |  | x |  | x |  |  |  |  |  |  |  |  |  |  |  |  |  |  |  | 1 | 2 |
| MIMAT0015041 | hsa-miR-1260b |  |  |  |  |  |  |  |  |  |  |  |  | x | x |  |  |  |  |  |  |  | 1 | 2 |
| MIMAT0004584 | hsa-let-7g-3p |  |  |  | x |  | x |  |  |  |  |  |  |  |  |  |  |  |  |  |  |  | 1 | 2 |
| MIMAT0004518 | hsa-miR-16-2-3p |  |  |  | x | x | x |  |  |  |  |  |  |  |  |  |  |  |  |  |  |  | 1 | 3 |
| MIMAT0000454 | hsa-miR-184 |  |  |  | x | x | x |  |  |  |  |  |  |  |  |  |  |  |  |  |  |  | 1 | 3 |
| MIMAT0004495 | hsa-miR-22-5p |  |  |  | x | x | x |  |  |  |  |  |  |  |  |  |  |  |  |  |  |  | 1 | 3 |
| MIMAT0009198 | hsa-miR-224-3p |  |  |  | x | x |  |  |  |  |  |  |  |  |  |  |  |  |  |  |  |  | 1 | 2 |
| MIMAT0015064 | hsa-miR-3184-5p |  |  |  | x |  |  |  |  |  |  |  |  |  |  |  |  |  |  |  |  |  | 1 | 1 |
| MIMAT0015071 | hsa-miR-3189-3p |  |  |  | x | x |  |  |  |  |  |  |  |  |  |  |  |  |  |  |  |  | 1 | 2 |
| MIMAT0015079 | hsa-miR-3195 |  |  |  | x |  |  |  |  |  |  |  |  |  |  |  |  |  |  |  |  |  | 1 | 1 |
| MIMAT0004703 | hsa-miR-335-3p |  |  |  | x | x | x |  |  |  |  |  |  |  |  |  |  |  |  |  |  |  | 1 | 3 |
| MIMAT0018003 | hsa-miR-3622a-5p |  |  |  | x |  |  |  |  |  |  |  |  | x |  |  |  |  |  |  |  |  | 2 | 2 |
| MIMAT0018116 | hsa-miR-3688-3p |  |  |  | x |  |  |  |  |  |  |  |  |  |  |  |  |  |  |  |  |  | 1 | 1 |
| MIMAT0018120 | hsa-miR-3691-5p |  |  |  | x |  |  |  |  |  |  |  |  |  |  |  |  |  |  |  |  |  | 1 | 1 |
| MIMAT0004813 | hsa-miR-411-3p |  |  |  | x | x | x |  |  |  |  |  |  |  |  |  |  |  |  |  |  |  | 1 | 3 |
| MIMAT0016881 | hsa-miR-4260 |  |  |  | x | x |  |  |  |  |  |  |  |  |  |  |  |  |  |  |  |  | 1 | 2 |
| MIMAT0016910 | hsa-miR-4278 |  |  |  | x |  |  |  |  |  |  |  |  |  |  |  |  |  |  |  |  |  | 1 | 1 |

**Table S7. continued**

| **MIMAT** | **miR name** | **R-SVM** | | | **RF** | | | **NB** | | | **LSVM** | | | **COX RS** | | | **LR** | | | **KNN** | | | **common**  **OUH** | **common**  **all cohorts** |
| --- | --- | --- | --- | --- | --- | --- | --- | --- | --- | --- | --- | --- | --- | --- | --- | --- | --- | --- | --- | --- | --- | --- | --- | --- |
|  |  | **O** | **M** | **D** | **O** | **M** | **D** | **O** | **M** | **D** | **O** | **M** | **D** | **O** | **M** | **D** | **O** | **M** | **D** | **O** | **M** | **D** |  |  |
|  |  | **30** | **12** | **21** | **107** | **42** | **68** | **38** | **15** | **26** | **29** | **11** | **20** | **109** | **37** | **65** | **10** | **4** | **10** | **10** | **6** | **10** |  |  |
| MIMAT0016853 | hsa-miR-4300 |  |  |  | x | x |  |  |  |  |  |  |  |  |  |  |  |  |  |  |  |  | 1 | 2 |
| MIMAT0016856 | hsa-miR-4303 |  |  |  | x | x |  |  |  |  |  |  |  |  |  |  |  |  |  |  |  |  | 1 | 2 |
| MIMAT0016867 | hsa-miR-4316 |  |  |  | x |  |  |  |  |  |  |  |  |  |  |  |  |  |  |  |  |  | 1 | 1 |
| MIMAT0016870 | hsa-miR-4319 |  |  |  | x |  |  |  |  |  |  |  |  |  |  |  |  |  |  |  |  |  | 1 | 1 |
| MIMAT0006778 | hsa-miR-516a-3p |  |  |  | x |  | x |  |  |  |  |  |  |  |  |  |  |  |  |  |  |  | 1 | 2 |
| MIMAT0002859 | hsa-miR-516b-5p |  |  |  |  | x |  |  |  |  |  |  |  |  |  |  |  |  |  |  |  |  | 0 | 1 |
| MIMAT0002848 | hsa-miR-518c-3p |  |  |  | x |  | x |  |  |  |  |  |  |  |  |  |  |  |  |  |  |  | 1 | 2 |
| MIMAT0005875 | hsa-miR-548j-5p |  |  |  | x |  | x |  |  |  |  |  |  |  |  |  |  |  |  |  |  |  | 1 | 2 |
| MIMAT0003248 | hsa-miR-583 |  | x |  | x | x | x |  |  |  |  | x |  |  |  |  |  | x |  |  |  |  | 1 | 6 |
| MIMAT0003255 | hsa-miR-588 |  |  |  | x |  | x |  |  |  |  |  |  |  |  |  |  |  |  |  |  |  | 1 | 2 |
| MIMAT0004819 | hsa-miR-671-3p |  |  |  | x |  | x |  |  |  |  |  |  |  |  |  |  |  |  |  |  |  | 1 | 2 |
| MIMAT0004554 | hsa-miR-7-2-3p |  |  |  | x |  | x |  |  |  |  |  |  |  |  |  |  |  |  |  |  |  | 1 | 2 |
| MIMAT0004923 | hsa-miR-875-3p |  |  |  | x |  | x |  |  |  |  |  |  |  |  |  |  |  |  |  |  |  | 1 | 2 |
| MIMAT0000441 | hsa-miR-9-5p |  |  |  | x | x | x |  |  |  |  |  |  |  |  |  |  |  |  |  |  |  | 1 | 3 |
| n/a | hsa-miRPlus-A1065 |  |  |  | x |  |  |  |  |  |  |  |  |  |  |  |  |  |  |  |  |  | 1 | 1 |
| n/a | hsa-miRPlus-J1003 |  |  |  | x |  |  |  |  |  |  |  |  |  |  |  |  |  |  |  |  |  | 1 | 1 |
| n/a | hsa-miRPlus-J98* |  |  |  | x |  |  |  |  |  |  |  |  |  |  |  |  |  |  |  |  |  | 1 | 1 |
| MIMAT0005866 | hsa-miR-1203 |  |  |  |  |  |  |  |  |  |  |  |  | x |  | x |  |  |  |  |  |  | 1 | 2 |
| MIMAT0002891 | hsa-miR-18a-3p |  |  |  |  |  |  |  |  |  |  |  |  | x |  | x |  |  |  |  |  |  | 1 | 2 |
| MIMAT0004763 | hsa-miR-488-3p |  |  |  |  |  |  |  |  |  |  |  |  | x | x | x |  |  |  |  |  |  | 1 | 3 |
| MIMAT0004773 | hsa-miR-500a-5p |  |  |  |  |  |  |  |  |  |  |  |  | x | x |  |  |  |  |  |  |  | 1 | 2 |
| MIMAT0002822 | hsa-miR-512-5p |  |  |  |  |  |  |  |  |  |  |  |  | x |  | x |  |  |  |  |  |  | 1 | 2 |
| MIMAT0015009 | hsa-miR-548t-5p |  |  |  |  |  |  |  |  |  |  |  |  | x |  |  |  |  |  |  |  |  | 1 | 1 |
| MIMAT0003219 | hsa-miR-555 |  |  |  |  |  |  |  |  |  |  |  |  | x |  | x |  |  |  |  |  |  | 1 | 2 |
| MIMAT0003270 | hsa-miR-602 |  |  |  |  |  |  |  |  |  |  |  |  | x | x | x |  |  |  |  |  |  | 1 | 3 |
| MIMAT0003275 | hsa-miR-607 |  |  |  |  |  |  |  |  |  |  |  |  | x |  | x |  |  |  |  |  |  | 1 | 2 |
| MIMAT0003276 | hsa-miR-608 |  |  |  |  |  |  |  |  |  |  |  |  | x |  | x |  |  |  |  |  |  | 1 | 2 |
| MIMAT0004804 | hsa-miR-615-5p |  |  |  |  |  |  |  |  |  |  |  |  | x |  | x |  |  |  |  |  |  | 1 | 2 |
| MIMAT0003284 | hsa-miR-616-5p |  |  |  |  |  |  |  |  |  |  |  |  | x |  | x |  |  |  |  |  |  | 1 | 2 |
| MIMAT0003311 | hsa-miR-641 |  |  |  |  |  |  |  |  |  |  |  |  | x | x | x |  |  |  |  |  |  | 1 | 3 |
| MIMAT0004559 | hsa-miR-181c-3p |  | x |  |  |  |  |  |  |  |  | x |  | x | x | x |  |  |  |  |  |  | 1 | 5 |

**Table S7. continued**

| **MIMAT** | **miR name** | **R-SVM** | | | **RF** | | | **NB** | | | **LSVM** | | | **COX RS** | | | **LR** | | | **KNN** | | | **common**  **OUH** | **common**  **all cohorts** |
| --- | --- | --- | --- | --- | --- | --- | --- | --- | --- | --- | --- | --- | --- | --- | --- | --- | --- | --- | --- | --- | --- | --- | --- | --- |
|  |  | **O** | **M** | **D** | **O** | **M** | **D** | **O** | **M** | **D** | **O** | **M** | **D** | **O** | **M** | **D** | **O** | **M** | **D** | **O** | **M** | **D** |  |  |
|  |  | **30** | **12** | **21** | **107** | **42** | **68** | **38** | **15** | **26** | **29** | **11** | **20** | **109** | **37** | **65** | **10** | **4** | **10** | **10** | **6** | **10** |  |  |
| MIMAT0009196 | hsa-miR-103-2-5p |  |  |  |  |  |  |  |  |  |  |  |  | x |  |  |  |  |  |  |  |  | 1 | 1 |
| MIMAT0002819 | hsa-miR-193b-3p |  |  |  |  |  |  |  |  |  |  |  |  | x | x | x |  |  |  |  |  |  | 1 | 3 |
| MIMAT0011156 | hsa-miR-2114-5p |  |  |  |  |  |  |  |  |  |  |  |  | x |  |  |  |  |  |  |  |  | 1 | 1 |
| MIMAT0011777 | hsa-miR-2277-3p |  |  |  |  |  |  |  |  |  |  |  |  | x |  |  |  |  |  |  |  |  | 1 | 1 |
| MIMAT0000079 | hsa-miR-24-1-5p |  |  |  |  |  |  |  |  |  |  |  |  | x | x | x |  |  |  |  |  |  | 1 | 3 |
| MIMAT0000717 | hsa-miR-302c-3p |  |  |  |  |  |  |  |  |  |  |  |  | x |  | x |  |  |  |  |  |  | 1 | 2 |
| MIMAT0005931 | hsa-miR-302e |  |  |  |  |  |  |  |  |  |  |  |  | x |  | x |  |  |  |  |  |  | 1 | 2 |
| MIMAT0015026 | hsa-miR-3153 |  |  |  |  |  |  |  |  |  |  |  |  | x |  |  |  |  |  |  |  |  | 1 | 1 |
| MIMAT0015028 | hsa-miR-3154 |  |  |  |  |  |  |  |  |  |  |  |  | x | x |  |  |  |  |  |  |  | 1 | 2 |
| MIMAT0015032 | hsa-miR-3158-3p |  |  |  |  |  |  |  |  |  |  |  |  | x |  |  |  |  |  |  |  |  | 1 | 1 |
| MIMAT0015061 | hsa-miR-3181 |  |  |  |  |  |  |  |  |  |  |  |  | x |  |  |  |  |  |  |  |  | 1 | 1 |
| MIMAT0015075 | hsa-miR-3191-3p |  |  |  |  |  |  |  |  |  |  |  |  | x |  |  |  |  |  |  |  |  | 1 | 1 |
| MIMAT0017996 | hsa-miR-3616-3p |  |  |  |  |  |  |  |  |  |  |  |  | x |  |  |  |  |  |  |  |  | 1 | 1 |
| MIMAT0018077 | hsa-miR-3657 |  |  |  |  |  |  |  |  |  |  |  |  | x |  |  |  |  |  |  |  |  | 1 | 1 |
| MIMAT0018106 | hsa-miR-3680-5p |  |  |  |  |  |  |  |  |  |  |  |  | x |  |  |  |  |  |  |  |  | 1 | 1 |
| MIMAT0018193 | hsa-miR-3919 |  |  |  |  |  |  |  |  |  |  |  |  | x |  |  |  |  |  |  |  |  | 1 | 1 |
| MIMAT0018198 | hsa-miR-3923 |  |  |  |  |  |  |  |  |  |  |  |  | x |  |  |  |  |  |  |  |  | 1 | 1 |
| MIMAT0015004 | hsa-miR-544b |  |  |  |  |  |  |  |  |  |  |  |  | x |  |  |  |  |  |  |  |  | 1 | 1 |
| n/a | hsa-miRPlus-K1303* |  |  |  |  |  |  |  |  |  |  |  |  | x |  |  |  |  |  |  |  |  | 1 | 1 |
| MIMAT0004812 | hsa-miR-548d-5p |  |  |  |  |  |  |  |  |  |  |  |  | x | x | x |  |  |  |  |  |  | 1 | 2 |
| MIMAT0016922 | hsa-miR-4291 |  |  |  |  |  |  |  |  |  |  |  |  | x | x |  |  |  |  |  |  |  | 1 | 2 |
| MIMAT0016846 | hsa-miR-4297 |  |  |  |  |  |  |  |  |  |  |  |  | x |  |  |  |  |  |  |  |  | 1 | 1 |
| MIMAT0016914 | hsa-miR-4283 |  |  |  |  |  |  |  |  |  |  |  |  | x |  |  |  |  |  |  |  |  | 1 | 1 |

**References**

1. Ritchie ME, Phipson B, Wu D, et al. limma powers differential expression analyses for RNA-sequencing and microarray studies. *Nucleic Acids Res*. 2015;43(7):e47. doi:10.1093/nar/gkv007

2. Cruz JA, Wishart DS. Applications of machine learning in cancer prediction and prognosis. *Cancer Inform*. Feb 11 2007;2:59-77.

3. Somorjai RL, Dolenko B, Baumgartner R. Class prediction and discovery using gene microarray and proteomics mass spectroscopy data: curses, caveats, cautions. *Bioinformatics*. Aug 12 2003;19(12):1484-91.

4. Breiman L. Random Forests. *Mach Learn*. 2001;45:5-32.

5. Burton M, Thomassen M, Tan Q, Kruse TA. Gene expression profiles for predicting metastasis in breast cancer: a cross-study comparison of classification methods. Comparative Study

Research Support, Non-U.S. Gov't. *TheScientificWorldJournal*. 2012;2012:380495. doi:10.1100/2012/380495

6. D'Aiuto F, Callari M, Dugo M, et al. miR-30e* is an independent subtype-specific prognostic marker in breast cancer. *Br J Cancer*. Jul 14 2015;113(2):290-8. doi:10.1038/bjc.2015.206

7. Dvinge H, Git A, Graf S, et al. The shaping and functional consequences of the microRNA landscape in breast cancer. *Nature*. May 16 2013;497(7449):378-82. doi:10.1038/nature12108

8. Curtis C, Shah SP, Chin SF, et al. The genomic and transcriptomic architecture of 2,000 breast tumours reveals novel subgroups. *Nature*. Apr 18 2012;486(7403):346-52. doi:10.1038/nature10983

9. Haunsberger SJ, Connolly NM, Prehn JH. miRNAmeConverter: an R/bioconductor package for translating mature miRNA names to different miRBase versions. *Bioinformatics*. Feb 15 2017;33(4):592-593. doi:10.1093/bioinformatics/btw660

10. Clopper C, Pearson E. The use of confidence or fiducial limits illustrated in the case of the binomial. *Biometrika*. 1934;26:404–413. doi:doi: 10.2307/2331986

11. Fuchs M, Beissbarth T, Wingender E, Jung K. Connecting high-dimensional mRNA and miRNA expression data for binary medical classification problems. *Comput Methods Programs Biomed*. Sep 2013;111(3):592-601. doi:10.1016/j.cmpb.2013.05.013

12. R Core Team. R: A language and environment for statistical computing. R Foundation for Statistical Computing, Vienna, Austria. URL <http://www.R-project.org/>. 2013;

13. Ritchie ME, Phipson B, Wu D, et al. limma powers differential expression analyses for RNA-sequencing and microarray studies. *Nucleic Acids Res*. Apr 20 2015;43(7):e47. doi:10.1093/nar/gkv007

14. Moller S, Jensen MB, Ejlertsen B, et al. The clinical database and the treatment guidelines of the Danish Breast Cancer Cooperative Group (DBCG); its 30-years experience and future promise. *Acta Oncol*. 2008;47(4):506-24. doi:10.1080/02841860802059259
